# Traditional healer-delivered point-of-care HIV testing versus referral to clinical facilities for adults of unknown serostatus in rural Uganda: a mixed-methods, cluster-randomised trial

**DOI:** 10.1016/S2214-109X(21)00366-1

**Published:** 2021-11

**Authors:** Radhika Sundararajan, Matthew Ponticiello, Myung Hee Lee, Steffanie A Strathdee, Winnie Muyindike, Denis Nansera, Rachel King, Daniel Fitzgerald, Juliet Mwanga-Amumpaire

**Affiliations:** Department of Emergency Medicine (R Sundararajan MD, M Ponticiello BS) and Center for Global Health (R Sundararajan, M H Lee PhD, D Fitzgerald MD), Weill Cornell Medicine, New York, NY, USA; Department of Medicine, University of California, San Diego, La Jolla, CA, USA (S A Strathdee PhD); Mbarara Regional Referral Hospital, Mbarara, Uganda (W Muyindike MD, D Nansera MD); Mbarara University of Science and Technology, Mbarara, Uganda (W Muyindike, D Nansera, J Mwanga-Amumpaire MD); Institute for Global Health Sciences, University of California, San Francisco, San Francisco, CA, USA (R King PhD)

## Abstract

**Background:**

HIV counselling and testing are essential to control the HIV epidemic. However, HIV testing uptake is low in sub-Saharan Africa, where many people use informal health-care resources such as traditional healers. We hypothesised that uptake of HIV tests would increase if provided by traditional healers. We aimed to determine the effectiveness of traditional healers delivering HIV testing at point of care compared with referral to local clinics for HIV testing in rural southwestern Uganda.

**Methods:**

We did a mixed-methods study that included a cluster-randomised trial followed by individual qualitative interviews among a sample of participants in Mbarara, Uganda. Traditional healers aged 18 years or older who were located within 8 km of the Mbarara District HIV clinic, were identified in the 2018 population-level census of traditional healers in Mbarara District, and delivered care to at least seven clients per week were randomly assigned (1:1) as clusters to an intervention or a control group. Healers screened their clients for eligibility, and research assistants confirmed eligibility and enrolled clients who were aged 18 years or older, were receiving care from a participating healer, were sexually active (ever had intercourse), self-reported not having received an HIV test in the previous 12 months (and therefore considered to be of unknown serostatus), and had not previously been diagnosed with HIV infection. Intervention group healers provided counselling and offered point-of-care HIV tests to adult clients. Control group healers provided referral for HIV testing at nearby clinics. The primary outcome was the individual receipt of an HIV test within 90 days of study enrolment. Safety and adverse events were recorded and defined on the basis of prespecified criteria. This study is registered with ClinicalTrials.gov, NCT03718871.

**Findings:**

Between Aug 2, 2019, and Feb 7, 2020, 17 traditional healers were randomly assigned as clusters (nine to intervention and eight to control), with 500 clients of unknown HIV serostatus enrolled (250 per group). In the intervention group, 250 clients (100%) received an HIV test compared with 57 (23%) in the control group, a 77% (95% CI 73–82) increase in testing uptake, after adjusting for the effect of clustering (p<0·0001). Ten (4%) of 250 clients in the intervention group tested HIV positive, seven of whom self-reported linkage to HIV care. No new HIV cases were identified in the control group. Qualitative interviews revealed that HIV testing delivered by traditional healers was highly acceptable among both providers and clients. No safety or adverse events were reported.

**Interpretation:**

Delivery of point-of-care HIV tests by traditional healers to adults of unknown serostatus significantly increased rates of HIV testing in rural Uganda. Given the ubiquity of healers in Africa, this approach holds promise as a new pathway to provide community-based HIV testing, and could have a dramatic effect on uptake of HIV testing in sub-Saharan Africa.

**Funding:**

US National Institute of Mental Health, National Institutes of Health.

## Introduction

HIV counselling and testing is a crucial entry point into care and an essential component of controlling the HIV epidemic.^1^ Uptake has been low in sub-Saharan Africa and other resource-poor settings.^2^ For example, in parts of rural Uganda, nearly 80% of sexually active men have not received an HIV test in the previous 12 months,^3^ and nearly half of men living with HIV are unaware of their status.^4^

Various community-based strategies have been evaluated in sub-Saharan Africa to increase HIV testing.^5^ Mobile clinic outreach in communities and home visits by health-care workers have shown some success,^6^ but have reduced effect among men and low rates of linkage to care.^7^ Community distribution of HIV self-testing kits has increased testing in some rural African populations;^8^ however, scale-up has been limited by low literacy, HIV-related stigma, desire for pre-test and post-test counselling, and poor linkage to care.^9^ Furthermore, these strategies have not been successfully brought to scale because they depend on novel infrastructure, which is expensive, logistically complex, and difficult to sustain.^10^

Collaboration with traditional healers has been proposed as another strategy to increase uptake of HIV testing.^11^ Traditional healers are ubiquitous, informal health-care providers. Communities in sub-Saharan Africa use healers instead of, or in addition to, biomedical services. Most individuals use both biomedical services and traditional healers, but healers tend to be visited more frequently than biomedical facilities.^12^ Previous research also indicates that adults living with HIV frequently receive care from healers.^13^ Some studies suggest that use of traditional healers is associated with delayed HIV diagnosis^14^ and reduced adherence to antiretroviral therapy (ART).^15^ A study in rural Mozambique showed that healers could refer clients to biomedical facilities for HIV testing; however, the referral process was impeded by stockouts of testing kits, health-care workers not accepting referrals from healers, and long distances to the health centre.^16^

Sensitive, specific, and easy-to-use oral swab HIV tests are now available for layperson use throughout sub-Saharan Africa.^17^ Used by individuals for self-testing alone or with a trusted person, they are approved by WHO^18^ and the Ugandan Ministry of Health.^19^

On the basis of our previous work in Uganda, we hypothesised that traditional healers would be willing and able to effectively deliver oral swab HIV tests to their clients who otherwise might not engage with HIV testing services.^20^ We designed a community-based point-of-care testing programme that was to be delivered by traditional healers directly to their clients using the approved oral swab HIV test. We aimed to evaluate the effectiveness of this novel HIV testing strategy compared with referral to clinical facilities for HIV testing in rural Uganda.

## Methods

### Study design and participants

We did a mixed-methods study that included a cluster-randomised trial, followed by individual qualitative interviews among a sample of participants. The parallel-arm, cluster-randomised study design was selected because we sought to evaluate an innovative approach to community-based HIV testing, and there would be high likelihood of cross-contamination if individual clients were randomly assigned at the time of seeking care.

This trial was conducted in Mbarara Township, a rural, agriculture-producing region of southwestern Uganda located about 270 km from the capital city of Kampala. Mbarara District’s largest government-supported HIV clinic provides free care to a catchment area of approximately 475 000 residents and is located in Mbarara Township. HIV prevalence is 7·9% in Mbarara, exceeding the national prevalence of 5·7%.^21^

Ugandan healers practise four distinct specialties: herbal medicine (herbalist), spiritual healing (spiritualists), prenatal care, labour, and delivery (birth attendants), and treatment of broken bones (bonesetters). Birth attendants are exclusively female and attend to female patients. Bonesetters are exclusively male, and treat patients of both genders. Spiritualists and herbalists are of both genders, and treat clients of both sexes. All four traditional healer specialties were included in this trial.

The unit of randomisation for the cluster-randomised trial was the traditional healer. We used a 2018 population-level census of all healers practising in Mbarara District to identify potential trial participants.^12^ We predetermined an 8 km one-way travel distance to the District HIV clinic as the geographical boundary within which to include healers as recruitment locations for this trial. This distance was established so that round-trip travel could be accomplished within a single day to receive HIV testing.

Healers were considered eligible for trial participation as cluster sites if they were aged 18 years or older; were located within 8 km of the Mbarara District HIV clinic; were identified in the 2018 population-level census of traditional healers in Mbarara District; and delivered care to at least seven clients per week. The final criterion excluded healers with average client volumes in the lowest quartile (six or fewer patients per week; the highest quartile was at least 28 patients per week), based on data from our 2018 census.^12^

Inclusion criteria for clients were age 18 years or older; receiving care from a participating healer; sexually active (ever had intercourse); self-report of not having received an HIV test in the previous 12 months; and not previously diagnosed with HIV infection. The Ugandan Ministry of Health^22^ recommends HIV testing for all people who have not received a test in the previous 12 months. On the basis of this guidance, we considered individuals who had not received a test within 12 months to be of unknown HIV serostatus. No changes to eligibility criteria were made after trial commencement.

Eligibility screening and informed consent took place at healer locations. All participants, including healers and their clients, provided written informed consent in the local language (Runyankole) to a Ugandan study research assistant.

No data safety monitoring committee was used for this trial. This study was approved by the institutional review boards of Mbarara University of Science and Technology, Ugandan National Council of Science and Technology, University of California, San Diego (La Jolla, CA, USA), and Weill Cornell Medicine (New York, NY, USA). The protocol is available in the appendix (pp 25–60).

### Randomisation and masking

Healers were randomly assigned (1:1) to the study intervention or control group, stratified by healer specialty (bonesetter, birth attendant, herbalist, and spiritualist). RS implemented the randomisation process. Each healer’s name was written on a card and then grouped by specialty. Cards for each specialty were drawn one at a time, in sequence. The healer named on the first card was assigned to the intervention group, and the healer named on the second card was assigned to the control group, and so on until all healers in that specialty were randomly assigned. The next specialty group of participating healers was then randomly assigned in the same manner.

Healers were provided with eligibility criteria for the trial and screened all clients receiving care at their practices during the enrolment period. If a client was determined to be potentially eligible, the healers would contact a study research assistant, who would arrive at the practice within 1 h to confirm eligibility and conduct study recruitment and enrolment procedures, if the client agreed. We recruited individual clients receiving care from participating healers in both study groups on a rolling basis. Screening and recruitment were continued at each healer site until target enrolment was reached. Blinding of participants and study staff was not possible due to the nature of the study intervention.

### Procedures

Healers from both study groups together attended a 1-day educational session on HIV transmission, HIV symptoms, HIV prevention, the role of ART, and Ugandan Ministry of Health HIV testing guidelines. This educational session was delivered by DN, a Ugandan infectious disease physician, and clinical director of the Mbarara District HIV clinic.

Referral to clinical facilities for HIV testing was defined as usual care for this trial based on data showing that African traditional healers refer their clients for HIV testing.^23^ For the purposes of this trial, we provided training and procedures to standardise the process of referral for HIV testing in the control group. Control group healers then attended another, separate 1-day training session provided by the Mbarara District HIV clinic nurse to be able to provide eligible clients with information about HIV transmission and the importance of voluntary HIV testing, and locations of HIV testing sites in Mbarara Township. The training session also described study eligibility criteria and study record-keeping procedures. Control group healers were provided with prepackaged envelopes containing a referral letter written in both English and Runyankole to give to eligible clients. The letter stated that the individual was participating in a study of HIV testing uptake and was interested in receiving voluntary HIV testing. After enrolment of eligible clients, healers were instructed to deliver HIV education and provide them with the referral envelope with information on nearby HIV testing locations. Participating clients were instructed to present the envelope at an HIV testing facility within 90 days.

Healers in the intervention group also attended another, separate 1-day training session provided by a Mbarara District HIV clinic nurse with instruction on pre-test and post-HIV test counselling, including role playing, practical training in administration of Oraquick HIV testing kits (Orasure Technologies, Bethleham, PA, USA), study eligibility criteria, and study record-keeping procedures. A Mbarara District HIV clinic nurse instructed healers on test kit usage, sample collection methodologies, and handwashing and hygiene measures during test administration. Oraquick HIV testing kits have been shown to be highly sensitive and specific in studies throughout sub-Saharan Africa.^17^ The testing kit is non-invasive and uses an oral swab sample to test for the presence of HIV antibodies. A control band identifies invalid tests, indicating when the kit has been used incorrectly. Tests are heat stable and require no refrigeration. During the training session, all intervention group healers practised delivering the test and interpreted test results correctly.

Once enrolled in the study at an intervention group site, clients were assigned a de-identified study number, which was placed on a recording form. The form was completely graphical, and reading competency was not required to complete it. For any clients testing positive on the point-of-care test, healers provided information about where to receive confirmatory testing and link to HIV care, if necessary. Research assistants delivered an individually wrapped Oraquick testing kit to the healer after enrolling an eligible client. Healers recorded whether the client accepted the test and, if so, the result of the point-of-care test. Research assistants collected these forms at the end of the treatment session. To maintain client confidentiality, research assistants did not view or validate point-of-care test results. Waste from Oraquick tests was removed by research assistants after the treatment session.

Clients were informed during enrolment that they would be contacted after 90 days by phone for an exit survey, and of the possibility of being recruited for a single in-depth, qualitative interview. All healers from both groups and a sample of their clients included in the trial were invited to participate in the single qualitative interview after trial completion. Healers were invited to participate following completion of enrolment at their practice. Key informants to interview were selected by RS among individual clients enrolled at healer locations, representing the range of trial outcomes, and diversity in sex, age, and marital status. Interviews were conducted by trained research assistants. Healer and client interviews contained similar questions, asking about their experiences participating in the study and factors relevant to the study’s primary and secondary outcomes. Interviews lasted approximately 60 min, were conducted in the local language (Runyankole), and were audio recorded. Interviews were transcribed and translated into English by the interviewing research assistant.

### Outcomes

Outcomes were assessed among individual clients of participating healers. The primary outcome was the individual receipt of an HIV test within 90 days of enrolment. In the control group, the primary outcome was assessed via self-report at time of 90-day follow-up phone call. For the intervention group, the participating healer recorded whether an HIV test was accepted by the client at the time of the study visit. If the HIV test was accepted and delivered, the healer recorded the result of the test. Secondary outcomes were new HIV diagnoses and linkage to HIV care within 90 days of enrolment for clients who were newly diagnosed. New HIV diagnoses and linkage to care were assessed via self-report during the 90-day follow-up phone call. For clients newly testing HIV positive, prespecified questions were posed to determine whether they had received confirmatory testing and initiated ART within 90 days of enrolment, if necessary. Clients who did not receive an HIV test were asked to describe reasons for not testing. Outcomes were defined before trial initiation and were not altered after study commencement. Safety and adverse events were recorded and defined on the basis of criteria presented in the protocol (appendix pp 35–36).

### Statistical analysis

The total number of clients screened at each cluster location during the enrolment period was determined on the basis of records kept by the healers indicating all patients who received treatment at their practices during that period. These records did not specify why an individual client might not have been eligible for participation. Therefore, data describing why screened clients were deemed ineligible are not reported.

We assumed an intracluster correlation coefficient of 0·2^24^ and predicted that 39% of patients in the control group would present for HIV testing at existing facilities.^3^ We hypothesised that the study intervention would increase the proportion of clients receiving an HIV test rate by 35% overall^5^ to 74% in the intervention group. We calculated that we would require 16 or more clusters, with a mean of 30 observations per cluster (total sample size of 480 clients) in order to have 80% power to detect this difference at an α level of 0·05.^24^ Target enrolment was set at 500 clients (250 per study group) divided equally among cluster sites in each group.

We summarised participant characteristics descriptively. For primary outcome analysis, we planned to use a multilevel logistic regression model, with individual clients in level 1 nested within healers in level 2, to calculate the odds ratio for receiving an HIV test in the intervention group compared with the control group, adjusting for the effects of clustering. When a regression model could not be created because of a zero in the denominator, we used Fisher’s exact test to assess for significant differences in the proportion of HIV testing between study groups.

For secondary outcomes of number of new HIV diagnoses and linkage to HIV care, we reported proportions of these outcomes in each study group and significance using Fisher’s exact test. We identified variables that predict HIV testing in the regression analysis post hoc. We evaluated the association between variables (appendix p 8) and the primary outcome of receiving an HIV test among control group clients, first identifying variables that were significant (two-sided p<0·05) based on univariate analysis and created a multivariate mixed-effects Poisson regression model accounting for covariates. In these models, we incorporated intercorrelation between clients from the same healer by including healers as random effects. We reported incident rate ratios and 95% CIs by fitting a multilevel Poisson regression model in the control group. Analyses were performed by MHL using Stata software (version 14).

Research assistants who conducted the post-trial interviews translated and transcribed them into English. English transcripts were reviewed by authors RS and MP, and analysed following a content analysis approach^25^ with the intent to identify content relevant to the study’s primary and secondary outcomes, and to explore participant experiences in the study. Illustrative quotes were selected to demonstrate themes in the interview data. The sample size for the qualitative data was guided by the concept of data saturation, whereby interviews no longer provide discordant or new information.

This study is registered with ClinicalTrials.gov, NCT03718871.

### Role of the funding source

The funder of the study had no role in the study design, data collection, data analysis, data interpretation, writing of the report, or decision to submit the paper for publication.

## Results

**Figure 1:**
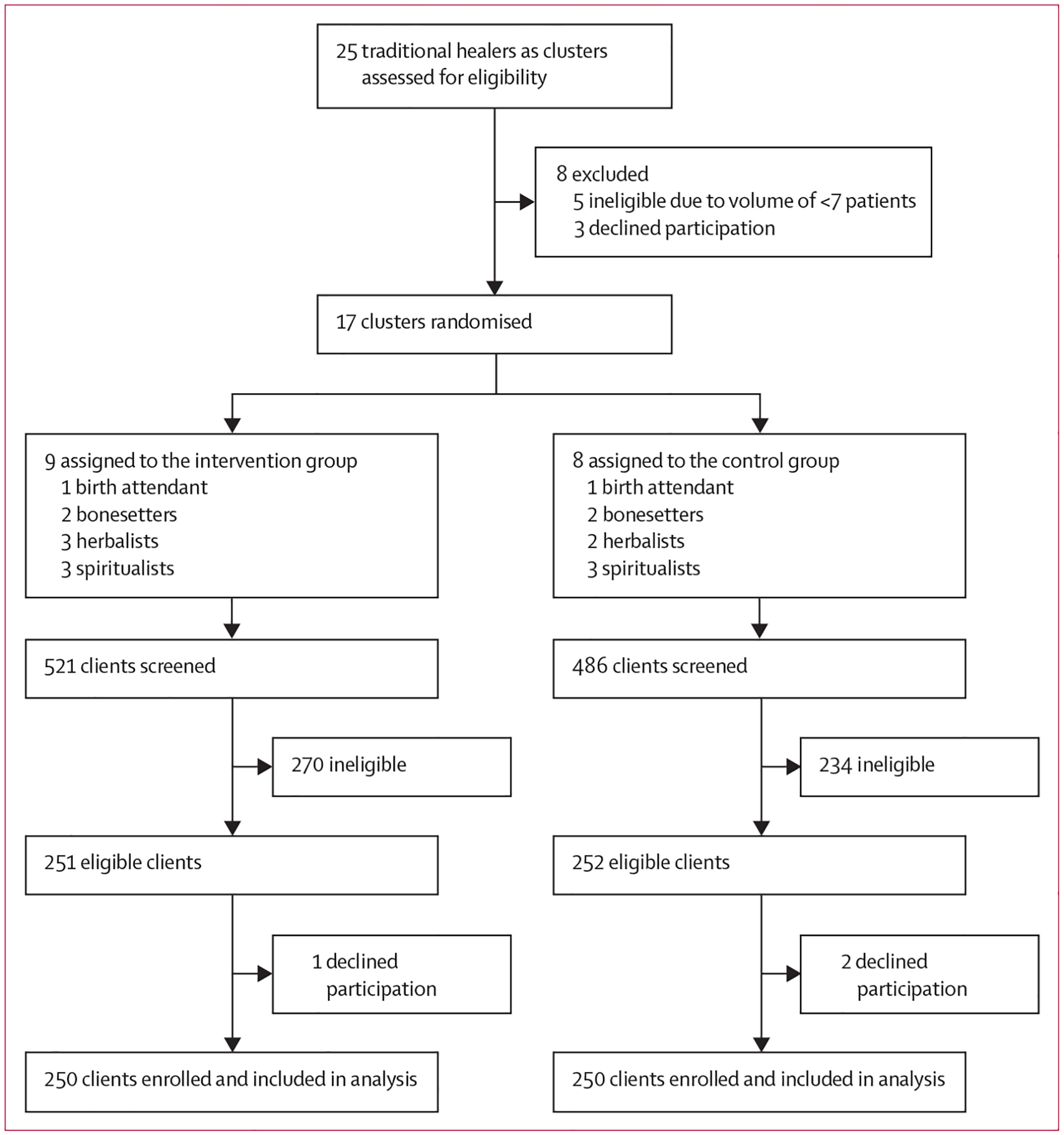
Trial profile

**Figure 2:**
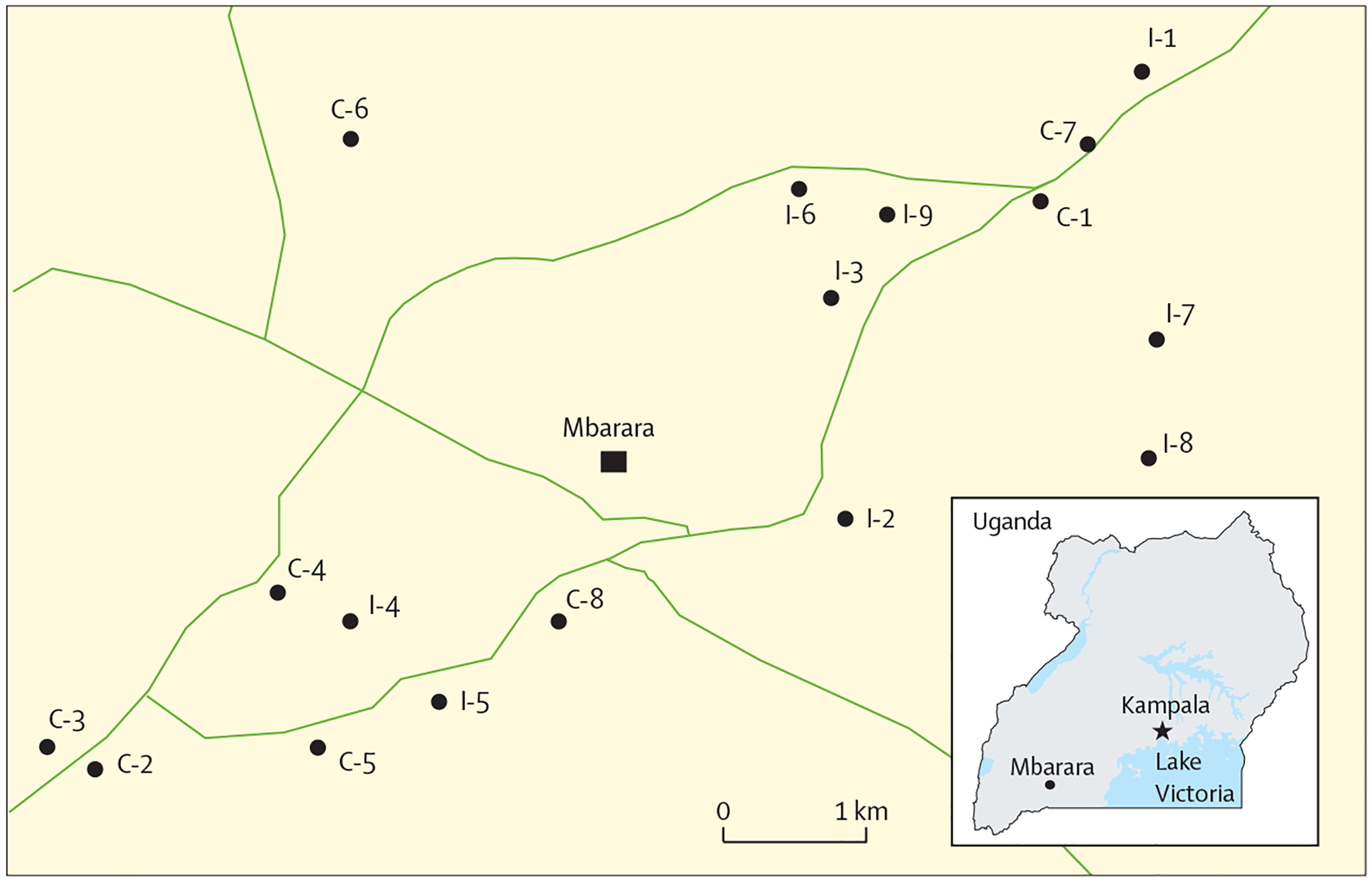
Location of traditional healer sites within 8 km radius of the Mbarara District HIV clinic included in the study C denotes control group sites. I denotes intervention group sites.

Our previous census identified 25 traditional healers practising within an 8 km distance from the Mbarara District HIV clinic.^12^ In July, 2019, these 25 sites were assessed for trial eligibility. Five were excluded due to self-reported weekly volume of fewer than seven clients, and three healers declined to participate because of scheduling conflicts with the mandatory 2-day training session preceding launch of the study (figure 1). On Aug 2, 2019, the remaining 17 healers were randomly assigned into two study groups (nine to intervention, eight to control; figures 1, 2). Between Aug 8, 2019, and Feb 7, 2020, 500 clients of unknown serostatus (250 per group) were enrolled at participating healer sites (figure 2). The enrolment period at each healer ranged from 3 weeks to 9 weeks. Cluster sizes ranged from 26 to 32 participants per healer, with a mean of 30 (SD 1·9) per cluster. In the intervention group, 248 (99%) of 250 clients were contacted at 90 days from enrolment; in the control group, all 250 (100%) were contacted. Baseline characteristics of traditional healers and individual clients are shown in table 1.

**Figure 3:**
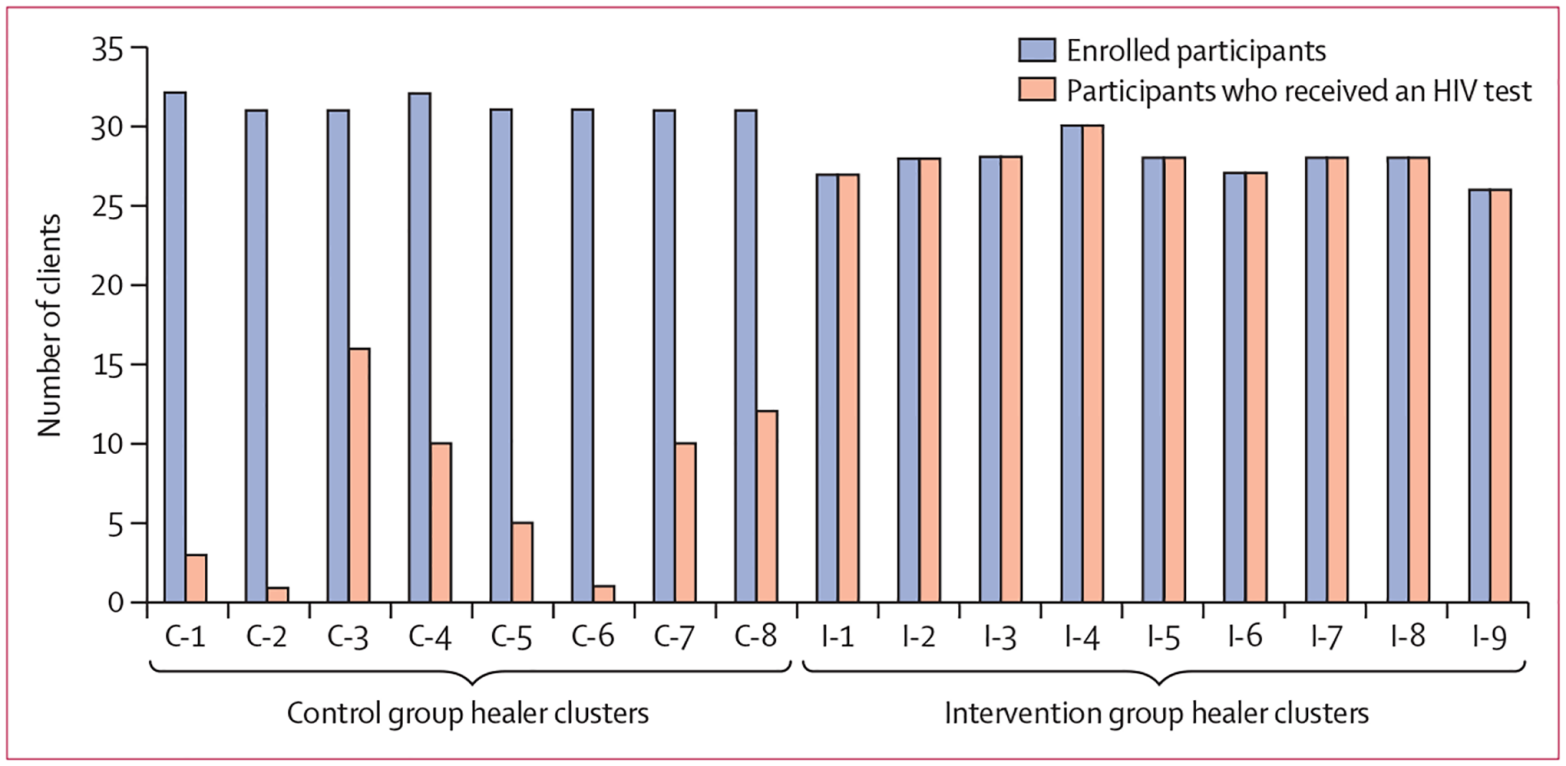
Numbers of individual clients enrolled and those who received an HIV test at each cluster location Cluster identification numbers C-1–8 on the left of the graph reflect control group sites. Clusters identification numbers I-1–9 on the right of the graph are intervention group sites.

In the intervention group, at 90 days from enrolment, all 250 (100%) participating clients received an HIV test, compared with 57 (23%) of 250 clients in the control group (p<0·0001; figure 3; table 2). The intervention increased the likelihood of receiving an HIV test 4·4 times, compared with usual care, with a difference in testing rates of 77% (95% CI 73–82), accounting for the effect of clustering at the level of the traditional healer. Because of the 100% HIV testing rate in the intervention group, the intracluster correlation coefficient was calculated for each study group, rather than for the study overall. In the intervention group, the intracluster correlation coefficient was 0. All clients received an HIV test, so there was no variation among the clusters in this group. In the control group, the intracluster correlation coefficient was 0·28 (95% CI 0·09–0·59), which was similar to the predicted value of 0·2 used in our sample size calculation.

In the intervention group, ten (4%) of 250 clients had a new HIV diagnosis compared with 0 of 250 in the control group (p=0·0018). Seven clients in the intervention group reported confirmatory HIV-positive test results, linkage to HIV care, and initiation of ART within 90 days of enrolment (table 3), compared with none in the control group (p=0·015). One client who tested HIV positive in the trial did not seek care within 90 days of enrolment. Two of the newly diagnosed clients were lost to follow-up at 90 days from enrolment, so secondary outcomes could not be confirmed; notably, these were the only participants lost to follow-up in the trial. Detailed enrolment and outcome data by group and by cluster are shown in the appendix (pp 15–16).

In the 90-day follow-up phone call, the 193 control group clients who did not receive an HIV test were asked to describe the major barriers to testing. Clients frequently named more than one barrier to receiving an HIV test at local clinics. Not having time to go for HIV testing (150 [78%] of 193 individuals) and insufficient funds to pay for transport to the HIV clinic (135 [70%]) were the most frequently cited reasons for not testing.

Three variables were significantly associated with the outcome of HIV testing in the univariate and multivariate analyses in the control group (table 4). Clients with elevated HIV risk scores were less likely to receive HIV testing than those with lower risk scores. Clients enrolled at bonesetter healers were also less likely to test than those enrolled at other healer specialties. Finally, younger clients were less likely to engage with formal HIV testing services than older clients. No safety or adverse events were reported.

**Figure 4:**
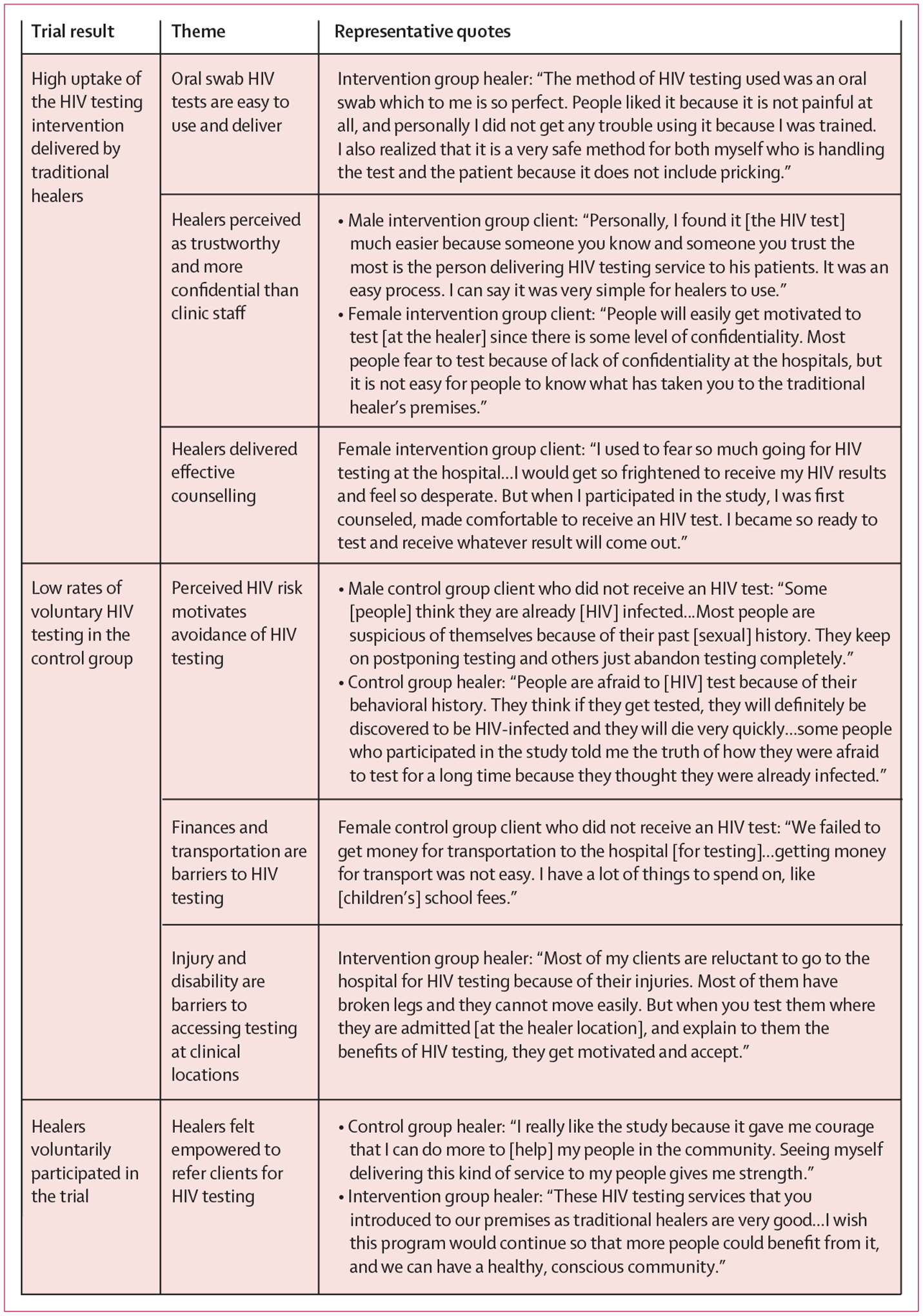
Summary of themes pertinent to trial results and quotes from exit interviews

After trial completion, 107 clients (67 from the control group and 40 from the intervention group) and all 17 healers were interviewed. Themes from exit interviews are shown in figure 4. In the intervention group, healer-delivered HIV testing was highly acceptable. Clients trusted healers and believed that the results of their HIV tests would remain confidential, and described healers as more confidential than clinic providers. Intervention group healers delivered effective pre-test and post-test counselling, and healers reported that Oraquick tests were easy to deliver and interpret. Healers participating in both study groups were enthusiastic to participate in this study, believing that they could have a positive effect on the health of their clients by supporting uptake of HIV testing, either through referral or delivery of HIV testing.

Control group clients faced numerous barriers in accessing facility-based HIV testing and avoided testing due to fear of testing positive. Clients who reported high-risk behaviours declined to seek HIV testing due to fear of testing positive. Insufficient funds to pay for transport to the clinics was also described as a barrier to receiving an HIV test. Control group clients enrolled at bonesetter healers reported that musculoskeletal injuries impeded mobility, which made it difficult to access voluntary HIV testing at clinic-based facilities.

## Discussion

This cluster-randomised trial shows the effectiveness of traditional healers in Mbarara delivering point-of-care HIV tests to their clients, referring those who test positive, and linking those who are newly diagnosed to HIV care. The intervention was associated with a significant increase in the rate of HIV testing, diagnosis, and linkage to care in rural Uganda compared with the control group. This strategy holds promise for large-scale implementation in Uganda and sub-Saharan Africa.

Our intervention demonstrated 100% uptake, higher than rates reported from previous community-based programmes in sub-Saharan Africa, including mobile outreach (97%), home-based HIV testing (80%), and distribution of HIV self-test kits (70%).^5^ Acceptance of healer-delivered testing can be attributed to the trusting relationships between healers and their clients. We, and others, have shown that healers are perceived as respected community members and trustworthy health-care providers.^26^ Additionally, our qualitative data illustrate that healers are believed to be more “confidential” than biomedical personnel, which might mitigate the stigma of HIV testing. Healer practices are not typically associated with HIV services, further reducing anticipated HIV-related stigma. Our intervention therefore leveraged the strength of client–healer relationships to deliver an acceptable and effective HIV testing programme, which might also explain high rates of linkage to care among individuals newly diagnosed with HIV.

Traditional medicine has been described as a “bottleneck” in the HIV cascade, delaying testing and entrance into services.^13^ Our intervention shows a means to shift the role of healers from bottlenecks to bottle-openers, expanding uptake of testing among hard-to-reach populations such as rural men, young adults, and individuals engaging in risky sexual behaviours. Previous studies in Africa have shown low rates of HIV testing among rural men and called for new strategies to increase their engagement in HIV testing.^27^ In our trial, healer-delivered testing effectively reached rural men: nearly half of all participants tested in the intervention group were men (116 [46%]), and four of the seven people newly diagnosed with HIV and linked to care were men. In the control group, we found low uptake of HIV testing among young adults and those engaging in risky sexual behaviours.^28^ Our qualitative data suggest that these participants might be “fearful” to receive HIV testing and that healers might be uniquely positioned to overcome this fear of testing.

Additionally, data from the control group confirm various structural barriers to accessing HIV testing, including time waiting at the testing facility, absence of transport, and high transport costs. These barriers have been well described in other studies in the region.^29^ We also found that clients in the control group enrolled at bonesetter healers had significantly lower rates of testing. Qualitative interviews showed that bonesetter clients had particular difficulty accessing clinic-based services because of physical injuries that impede mobility. Bringing HIV testing resources into communities will make access to testing less burdensome for patients and is congruent with differentiated service initiatives.^30^

A traditional healer is located in almost every village in sub-Saharan Africa. Others have discussed inclusion of African healers in HIV care,^11,16^ but to our knowledge this is the first rigorous randomised trial showing their effectiveness in delivering HIV testing to their clients. We showed that they can effectively deliver point-of-care HIV testing and refer individuals testing positive for ART initiation. Given the ubiquity of healers in Africa, this approach holds promise as a new pathway to access populations at high risk of HIV and provide community-based HIV services. This strategy could have a dramatic impact on HIV prevention and care in Africa, and on public health more broadly because it could be expanded to facilitate diagnosis via self-testing of infectious diseases, such as hepatitis C, malaria, and COVID-19.

This study has some limitations. This was not a large-scale cost-effectiveness trial. Our goal was to evaluate the effectiveness of a novel implementation strategy. We believe that this programme will be cost-effective, as minimal infrastructure was needed, and we did not advertise delivery of HIV testing or recruit clients to healer practices during enrolment. However, future studies are needed to evaluate the cost of the programme. Because we excluded healers with the lowest client volume, results might not be generalisable to these smaller practices. Large-scale implementation studies are needed to address these questions and adapt the intervention to local contexts. Further research is also needed to assess how individual characteristics of traditional healers might influence intervention delivery and uptake. Study outcomes in the control group were self-reported and therefore could be subject to social-desirability bias, although over-reporting of HIV testing rates would be expected if this were the case. Although our linkage to care rates were high in the intervention group, the overall sample of people living with HIV was small and larger studies are needed to evaluate linkage to care through referrals from healers. Finally, we provided a 2-day training session to healers in the control group and acknowledge that this itself was an intervention; the differences between intervention and control groups would have probably been even greater had we not provided education on the HIV clinic referral process.

In conclusion, we evaluated the effectiveness of a novel HIV testing programme in a cluster-randomised trial. Delivery of point-of-care HIV tests by traditional healers to adults of unknown serostatus increased uptake of testing more than four times, compared with referral to existing resources. This intervention was considered to be highly acceptable by clients and healers. This approach holds promise to expand HIV testing among hard-to-reach populations in HIV endemic regions of sub-Saharan Africa.

## Supplementary Material

1

## Figures and Tables

**Table 1:** Baseline characteristics of traditional healer clusters and individual participants

	Intervention group	Control group
**Traditional healers**		
Number of healers	9	8
Healer specialty		
Birth attendant	1 (1%)	1 (13%)
Bonesetter	2 (2%)	2 (25%)
Herbalist	3 (33%)	2 (25%)
Spiritualist	3 (33%)	3 (38%)
Age, years	40 (35–51)	47 (35–52)
Sex		
Female	4 (44%)	2 (25%)
Male	5 (56%)	6 (75%)
Primary school education or less	3 (33%)	2 (25%)
Patients per week	15 (7–20)	7 (7–12)
Healer distance to Mbarara District HIV clinic, km	4·3 (3·9–7·0)	5·3 (4·9–7·6)
**Clients**		
Number of participants	250	250
Age, years	29 (23–8)	30 (24–39)
Sex		
Female	134 (54%)	145 (58%)
Male	116 (46%)	105 (42%)
Primary school education or less	143 (57%)	139 (56%)
Christian religion	237 (95%)	218 (87%)
Married	142 (57%)	164 (66%)
Household size	5·0 (4·0–7·0)	4·5 (3·0–6·0)
Monthly household income, Ugandan Shillings	100 000 (50 000–300 000)	100 000 (50 000–200 000)
HIV risk score	2 (1–3)	2 (1–3)
HIV knowledge score	14 (12–15)	14 (12–15)
HIV stigma score	1.0 (0·6–1·3)	1·0 (0·4–1·2)
Ever received an HIV test	222 (89%)	218 (87%)
Months since last HIV test among those ever tested	26 (19–38)	34 (22–46)
Enrolled from specialty		
Birth attendant	28 (11%)	32 (13%)
Bonesetter	55 (22%)	62 (25%)
Herbalist	86 (34%)	63 (25%)
Spiritualist	81 (32%)	93 (37%)

Data are n (%) or median (IQR) unless otherwise stated.

**Table 2:** Summary of study groups and trial outcomes

	Intervention group (n=250)	Control group (n=250)	p value
Duration of recruitment at cluster, weeks	5 (4–5)	4 (3–5)	0·16
Clients enrolled/clients screened (%)	250/521 (48%)	250/486 (51%)	0·17
Clients tested for HIV	250 (100%)	57 (23%)	<0·0001
Clients newly diagnosed with HIV	10 (4%)	0	0·0018
HIV-positive clients linked to care within 90 days of enrolment*	7/10 (70%)	NA	NA

Data are median (IQR) or n (%) unless otherwise stated. NA=not applicable.

* Linkage to HIV care could not be established with two newly diagnosed HIV-positive clients who were lost to follow-up at 90 days.

**Table 3:** Summary characteristics of the participants newly diagnosed with HIV*

	Clients newly diagnosed with HIV (n=10)
Sex	
Female	6
Male	4
Age, years	37 (29–44)
Previously received an HIV test	8
Previous HIV tests (for those previously tested)	3 (2–4)
Months since last HIV test (for those previously tested)	30 (21–33)
Linked to HIV care within 90 days of enrolment†	7

Data are n or median (IQR).

* All participants were from the intervention group.

† Linkage to HIV care could not be established with two newly diagnosed HIV-positive clients who were lost to follow-up at 90 days.

**Table 4:** Results from univariate and multivariate analysis related to outcome of receiving an HIV test at local medical facilities

	Univariate incidence rate ratios	p value	Multivariate incidence rate ratios	p value
Age, years	1·02 (1·00–1·04)	0·0034	1·02 (1·00–1·03)*	0·013
Male vs female sex	0·86 (0·50–1·48)	0·58	NA†	NA†
Primary school education or less vs secondary or higher education	0·75 (0·42–1·34)	0·32	NA†	NA†
Married *vs* not married	0·90 (0·56–1·44)	0·66	NA†	NA†
HIV risk score	0·73 (0·62–0·85)	<0·0001	0·75 (0·66–0·86)‡	<0·0001
HIV knowledge score	1·07 (0·99–1·16)	0·10	NA†	NA†
HIV stigma score	1·19 (0·56–2·51)	0·65	NA†	NA†
Enrolled at bonesetter vs birth attendant, herbalist, or spiritualist	0·11 (0·07–0·18)	<0·0001	0·12 (0·08–0·19)	<0·0001

95% CI values are in parentheses. NA=not applicable.

* Per year increase in age.

† Multivariate analysis conducted for variables that were significant in the univariate analysis.

‡ Per 1-point increase in risk score.

## Research in context

### Evidence before this study

Our previous work in Uganda suggested that a traditional healer-based intervention could be an effective means to encourage uptake of HIV testing among populations that might otherwise be difficult to reach. We searched PubMed for research articles published between Jan 1, 2000, and Dec 31, 2020, using the broad search terms “HIV”, “HIV testing”, “traditional healers”, “faith healers”, and “sub-Saharan Africa”. We applied no language restrictions. Among the articles identified, we sought observational studies, formative qualitative studies, cluster-randomised trials, and systematic reviews. Several journal articles presented information about the patients who receive care from traditional and faith healers in sub-Saharan Africa, describing low rates of HIV testing, low CD4 cell count at entry into care, and poor adherence to antiretroviral therapy (ART). In 2017, Moshabela and colleagues described traditional healers as a “bottleneck” in the HIV cascade, with qualitative data showing delayed and disrupted care among patients living with HIV. Many articles also described the status and credibility that traditional healers have in their communities, suggesting that their influence could be leveraged to encourage uptake of HIV testing among the populations they serve, who might be difficult to reach through conventional programmes. Some have suggested integrating traditional healers and other informal providers into the HIV continuum of care. Studies have indicated that healers are both willing and able to participate in HIV education and counselling, and referral of patients to HIV testing facilities. In 2013, Audet and colleagues showed that an educational intervention could effectively increase the numbers of patients traditional healers referred for HIV testing; however, less than 4% of patients referred actually received HIV testing at the medical clinics. No systematic or cluster-randomised trial has been done to test whether traditional healers can effectively deliver HIV testing to their clients.

### Added value of this study

Despite major international efforts to increase uptake of HIV testing in sub-Saharan Africa, uptake has been suboptimal, particularly among populations that receive care from traditional healers. Our cluster-randomised trial shows that point-of-care delivery of HIV testing by traditional healers increased the likelihood of receiving an HIV test 4·4 times compared with usual care. In addition, clients newly diagnosed with HIV by traditional healers had significantly higher rates of ART initiation than those in the control group. These findings speak to the potential of incorporating traditional healers into the HIV continuum of care to increase rates of HIV testing and improve HIV-related outcomes in sub-Saharan Africa.

### Implications of all the available evidence

Our findings suggest, when added to the previous literature, that traditional healers can play a central part in expanding uptake of HIV testing among their clients. Although some studies have identified traditional healers as key stakeholders in the HIV care continuum, none have worked directly with healers to scale-up delivery of HIV testing. The success of this intervention, coupled with the ubiquity of traditional healers in sub-Saharan Africa, presents a promising opportunity to reach hard-to-reach populations that have suboptimal engagement with HIV services.
