# Supplementary material for: Traditional healer-delivered point-of-care HIV testing versus referral to clinical facilities for adults of unknown serostatus in rural Uganda: a mixed-methods, cluster-randomised trial
Source: Lancet Glob Health. Author manuscript; Available in PMC 2021 Nov 2. (PMC8562591; doi:10.1016/S2214-109X(21)00366-1)
Supplement: 1 [file NIHMS1749771-supplement-1.pdf]

THE LANCET
Global Health

Supplementary appendix

This appendix formed part of the original submission and has been peer reviewed. We post it as supplied by the authors.

Supplement to: Sundararajan R, Ponticiello M, Lee MH, et al. Traditional healer-delivered point-of-care HIV testing versus referral to clinical facilities for adults of unknown serostatus in rural Uganda: a mixed-methods, cluster-randomised trial. *Lancet Glob Health* 2021; **9:** e1579–88.

Supplementary Appendix

This appendix has been provided by the authors to give readers additional information about their work.

Traditional healer-delivered point-of-care HIV testing versus referral to clinical facilities for adults of unknown serostatus in rural Uganda: a mixed-methods, cluster randomized trial.

Supplementary Materials

CONTENTS:

Trial design details – page 3

Trial setting details – page 4

Trial population and eligibility criteria – page 5

Screening and informed consent – page 6

Method of randomization – page 7

Study measures – page 8

Detailed study procedures – page 10

Qualitative data collection methods and analysis – page 12

Trial sample size calculations – page 13

Statistical analysis details – page 14

Additional trial results – page 15

Additional qualitative results – page 17

Figure S1: Summary of enrollment and outcomes by study arm and cluster – page 18

Table S1: Study outcomes shown by study arm and cluster site – page 19

Table S2: Characteristics of participants newly diagnosed with HIV infection – page 20

Table S3: Disaggregated results from KQ-18 HIV knowledge questionnaire – page 21

Table S4: Disaggregated results from HIV Risk Score – page 22

References – page 23

Study protocol – page 25

Trial Design Details

This was a Hybrid Type 1 effectiveness-implementation trial[1], evaluating a novel strategy for delivering HIV tests to adults of unknown serostatus, measuring related clinical outcomes, and gathering data on contexts pertinent to intervention implementation. Parallel-arm, cluster randomized study design was selected as we sought to evaluate an innovative approach to deliver HIV testing, and there would be high likelihood of cross-contamination if individual patients were randomized at the time of seeking care.

Trial setting

Mbarara District is located ~270 kilometers from the capital city of Kampala. It is a rural, agriculture-producing region with approximately 475,000 residents. The primary formal health facility in this District is Mbarara Regional Referral Hospital (MRRH), a tertiary referral center and teaching hospital for Mbarara University of Science and Technology. MRRH runs the Immune Suppression Syndrome (ISS) clinic, the District's largest government-supported HIV clinic, which provides free HIV care to residents in Mbarara District. The ISS clinic, and other HIV testing facilities, are located in Mbarara Township.

In Uganda, as throughout sub-Saharan Africa, traditional healers are frequently utilized for healthcare needs[2]. We have shown that healers may be preferred due to ease of access and perceived lower cost, compared with biomedical facility use[3]. Healers in Uganda practice four distinct specialties: herbal medicine (herbalist), spiritual healing (spiritualists), prenatal care, labor and delivery (birth attendants), and treatment of broken bones (bonesetters). Birth attendants are exclusively female and attend to female patients. Bonesetters are exclusively male, and treat patients of both genders. Spiritualists and herbalists are of both genders, and treat patients of both genders.

Trial population and eligibility criteria

In 2018, we completed a population-level census of all traditional healers practicing in Mbarara District[4]. We predetermined an 8-kilometer one-way travel distance to the ISS HIV clinic as the geographic boundary within which to include traditional healers as recruitment locations for this trial. This distance was established as a walking distance to one or more HIV testing resource are centralized. Twenty-five traditional healers were identified within this area.

For the current trial, traditional healers were considered eligible for participation as cluster sites if they were 1) were aged 18 years or older; 2) were located within eight kilometers of the ISS clinic; 3) were identified in the 2018 population-level census of traditional healers in Mbarara District; and 4) delivered care to at least 7 patients per week. The final criterion excluded healers with average patient volumes in the lowest quartile, based on data from our 2018 census[4].

We recruited individual patients receiving care from participating traditional healers. Inclusion criteria for individual participants were: 1) aged 18 years or older; 2) receiving care from participating traditional healer; 3) sexually active (ever had intercourse); 4) self-report of not having received an HIV test in the prior 12 months; and 5) not previously diagnosed with HIV-infection. Ugandan Ministry of Health[5] and Center for Disease Control and Prevention recommend HIV testing for all people who have not received a test in the prior 12 months[6]. Based on these guidelines, we considered those who had not received a test within 12 months to be of “unknown HIV serostatus”.

Participants were recruited in both study arms from August 2019 – February 2020 on a rolling basis. There were no changes to eligibility criteria for healers or participants after trial commencement.

Screening and informed consent

Healers were provided with eligibility criteria for the trial, and performed eligibility screening for all patients receiving care at their practices during the enrollment period. If a patient was determined to be potentially eligible, the healers would contact a study research assistant, who would arrive at the practice within one hour to confirm eligibility and conduct study recruitment and enrollment, if the patient agreed.

Study participants received written informed consent in the local language (Runyankole), delivered by a Ugandan Research Assistant. They were informed at the time of study enrollment that they would be contacted after 90 days by phone for an exit survey, and of the possibility of an in-depth interview. Screening and recruitment were continued at each healer site until target enrollment of ~30 patients was reached. Among eligible participants, one person in the control arm and two people in the intervention arm declined to participate in the study citing lack of time needed to complete enrollment procedures. No participants withdrew from the study.

Individual trial participants received 10,000 Ugandan Shillings (~$2) worth of laundry soap as compensation for their time. No remuneration was provided to traditional healers for participating in the trial.

Method of randomization

We employed stratified randomization to divide healers into study arms, accounting for healer specialty (bonesetter, birth attendant, herbalist, spiritualist). The decision to apply stratified randomization based on healer specialty was informed by our prior work in the region, which illustrates a traditional healer's specialty accounts for differences between patients who receive care at these practice locations[4]. Specifically, our preliminary work suggested that some types of healers see patients who are less likely to know their HIV status, compared with other healer specialties. For example, traditional birth attendants provide care for pregnant and post-partum women, many of whom have received HIV testing as part of antenatal care.

Author RS implemented the randomization process. Each healer's name was written on a card, and then grouped by specialty. Cards for each specialty were drawn one-at-a-time, in sequence. The healer named on the first card was assigned to the intervention arm, and the healer named on the second card was assigned to the control arm, and so on until all healers in that specialty were randomized. The next specialty group of participating healers was then randomized in the same fashion. Gender was not considered in the randomization processes of healers into study arms. As all traditional healer practices were recruited from within an 8-kilometer radius from Mbarara Township, practice location was not considered as a variable for the randomization process.

Study measures

Cluster-level measures included traditional healer gender, age, specialty, total volume of adult patients seen during the study recruitment period, total number of study patients enrolled, and length of enrollment. We also measured one-way overland distance from each cluster site to the ISS HIV clinic. Individual baseline characteristics were collected at time of study enrollment, and include gender, age, household size, household income, highest level of education, and personal history of HIV testing. We also linked participants to the cluster location where they were enrolled. We administered questions from validated scales to assess HIV knowledge, HIV risk, and HIV-related stigma.

We included the KQ-18 HIV knowledge scale questions, which included 18 questions to evaluate HIV knowledlge[7]. We created an HIV risk scale that contained five questions about different high risk sexual behaviors, with high scores indicating higher acquisition risk, guided by data from previously published HIV risk scales utilized in this region[8-10]. We included the community-level HIV stigma scale questions from the PopART trial (HPTN 071) to evaluate a participant's stigmatizing beliefs about people living with HIV[11].

These scales have not been previously translated to the local language of this region. Therefore, author JMA (fluent in both English and Runyankole) translated scale questions directly from English to Runyankole. Two Ugandan research assistants, also fluent in Runyankole and English, used the Runyankole translation and translated these back into English. The back-translation process was performed to verify preservation of meaning from original, English scale questions.

The primary outcome for this study was receipt of an HIV test within 90 days of study enrollment. In the control arm, this was assessed via self-report at time of 90-day follow up

phone call. If no HIV test was received, participants were asked to specify the major barriers to tested. For intervention arm, the participating healer recorded whether an HIV test was accepted by the participant at the time of the study visit. If the HIV test was accepted and delivered, the healer recorded the result of the test. Secondary trial outcomes were assessed via self-report during the 90-day follow up phone call. For participants newly testing HIV-positive, questions were posed to determine if they had received confirmatory testing, and initiated ART medication, if necessary.

Detailed study procedures

Following randomization, healers were informed of which study arm they were assigned to and asked to participate in a two-day training session prior to launch of the study. In the control arm, healers provided HIV education and referral for HIV testing at existing resources. In the intervention arm, healers were trained to deliver pre- and post-HIV test counseling and point-of-care HIV testing.

Both study arms: In June 2019, healers from both study arms attended a one-day educational session on HIV transmission, symptoms, prevention, the role of anti-retroviral therapies, and Ugandan Ministry of Health HIV testing guidelines. This educational session was delivered by author DN, a Ugandan infectious disease physician, and Clinical Director of HIV services at ISS clinic.

Control arm: Referral to HIV testing was defined as usual care for this trial based on data from sub-Saharan Africa illustrating that traditional healers refer their patients for HIV testing[12,13]. For the purposes of this trial, we delivered training and procedures to standardize the process of referral for HIV testing in the control arm.

First, healers in the control arm attend a separate one-day training to provide eligible participants with information about HIV transmission and the importance of voluntary HIV testing, and locations of HIV testing sites in Mbarara Township. This educational session provided a shared knowledge base about the importance of HIV testing, and established a standard process for clinic referrals. Control arm healers were provided with pre-packaged envelopes, containing a referral letter written in both English and Runyankole. The letter stated that the bearer of the envelope was participating in a study of HIV testing uptake, and was interested in receiving voluntary HIV testing. After an eligible patient was enrolled in the study, healers were instructed to deliver HIV education, and provide them with a referral envelope with information on nearby HIV testing locations. Participants were instructed to present the

envelope at an HIV testing facility within 90 days. The training session also described study eligibility criteria and study record-keeping procedures.

Intervention arm: On a separate day, healers randomized to the intervention arm attended a one-day training program which focused on pre- and post-HIV test counseling including role playing, practical training in performing of Oraquick©, study eligibility criteria, and study record-keeping procedures. Oraquick HIV testing kits have been shown to be highly sensitive and specific in studies through sub-Saharan Africa[14-16]. The testing kit is non-invasive, and uses an oral swab sample to test for presence of HIV antibodies. A control band identifies invalid tests, indicating when the kit has been used incorrectly. These tests are heat-stable, and do not require refrigeration. During the training session, all intervention arm healers practiced delivering the test, and interpreted test results correctly. Training was led by a clinical nurse from ISS clinic who instructed healers on test kit utilization, sample collection methodologies, as well as handwashing and hygiene measures during test administration.

Healers were also oriented to study record-keeping. Once enrolled in the study at an intervention arm site, participants were assigned a de-identified study ID, which was placed on a recording form. The form was completely graphical, and did not require reading competency in order to complete it. For any participants testing positive on the point-of-care test, healers provided information on where to receive confirmatory testing and link to HIV care if necessary. Research assistants delivered an individually-wrapped Oraquick testing kit to the healer after enrolling an eligible patient. Healers recorded whether the participant accepted the test, and if so, the result of the point-of-care test. These forms were collected by study research assistants at the end of the treatment session. In order to maintain patient confidentiality, research Assistants did not view the point-of-care test results directly. Waste from Oraquick tests was also removed by research assistants at the end of the treatment session.

Qualitative Data Collection and Analysis

Qualitative interview methods

Data collection involved a single qualitative interview. All participating healers were invited to participate following completion of enrollment at their practice location. Key informants were selected among individual participants enrolled at healer locations, representing the range of study outcomes, and diversity in gender, age, and marital status. The interviews for both healers and their clients contained similar questions, asking about their experiences participating in the study, and factors relevant to the study's primary and secondary outcomes. Interviews lasted approximately 60 minutes, were conducted in the local language by Ugandan Research Assistants, and were audio recorded with permission. Participants who agreed to be interviewed received 10,000 Ugandan Shillings (~$2) worth of laundry soap as compensation for their time.

Qualitative sample size

Sample size for the qualitative data was guided by the concept of data saturation, whereby interviews no longer provide discordant or new information[17]. Data saturation was reached after 124 total interviews (N=107 with participants in both study arms, N=17 with healers).

Qualitative data analysis

Qualitative interview data was translated and transcribed into English by the research assistant who conducted the interview. English transcripts were reviewed by authors RS and MP, and analyzed following a content analysis approach[18,19]. Transcripts were coded for content relevant to the study's primary and secondary outcomes, as well as participant perspectives on the overall study. Illustrative quotes were selected to demonstrate patterns in the interview data, and are shown in the manuscript as Table 3.

Trial sample size calculation

Sample size was calculated to ensure adequate power to test the hypothesis that healer-delivered point-of-care HIV tests would increase the proportion of HIV testing among adults of unknown serostatus. There are scant data on uptake of HIV testing among populations who receive care at traditional healers. However, we predicted that the rate of HIV testing would be 39% for the control arm, based on population-level estimates from the Ugandan Ministry of Health[20].

We assumed intracluster correlation coefficient (ICC) estimate = 0.2 based on prior cluster randomized trials conducted in East Africa[21]. We predicted that the intervention would increase the proportion of participants receiving an HIV test rate from 39% in the control to 74% in the intervention arm (an overall increase of 35%). This estimate effect of the intervention was based on prior findings of community-based HIV testing in sub-Saharan Africa[22]. Using methods for calculating sample size for stratified cluster randomized trials[21], assuming ICC of 0.2, we calulated that we would need to include 16 clusters total, with an average of 30 observations per cluster (total sample size of 480 participants), to have 80% power to detect this difference at alpha = 0.05. Target enrollment number for clients was therefore set at 250 per study arm, divided equally among cluster sites in each study arm.

Statistical Analysis Details

For Table 1 in the manuscript, p-values indicating statistically significant differences between participants in each arm were calculated accounting for the effects of clustering at the level of the traditional healer. For primary outcome analysis, we planned to use a multi-level logistic regression model to calculate the odds ratio for HIV testing in the intervention compared with the control arms, adjusting for the effects of clustering. However, given that the intervention arm showed 100% uptake, a regression model comparing the primary outcomes results could not be created with a zero in the denominator. We therefore report a p-value using Fisher's exact test for difference in testing rates between study arms.

For secondary outcomes of number of new HIV diagnoses, and linkage to HIV care, we reported proportions of these outcomes in each study arm, and statistical significance using a Fisher's exact test. We considered variables that predict HIV testing among control arm only, given that uptake was 100% in the intervention. We reported proportions and 95% confidence intervals by fitting a multi-level Poisson regression in the control arm. Using univariate logistic models, we measured the association between variables and the primary outcome of receiving an HIV test. Based on results of the univariate analysis, we created a mixed-effects Poisson regression model accounting for covariates. In these models, we incorporated intercorrelation between participants from the same healer by including healers as random effects. In this mixed effect model, categorical variables were transformed into binary indicators for analysis. In the case of traditional healer specialty, participants enrolled at other three categories (birth attendant, herbalist, spiritualist) showed similar HIV testing rates (35%, 29%, 28%) compared to those enrolled at bone setters (3%). Therefore, the category of bonesetter was used as a binary indicator to distinguish those enrolled at bonesetter practices vs enrolled at other types of healers. Analyses were performed by author MHL using Stata software (version 14).

Additional Trial Results

The total number of patients screened at each cluster location during the enrollment period was determined based on records kept by the healers indicating the total patients who received treatment at their practices during the enrollment period. We did not require participating healers to record why an individual patient may not have been eligible for participation. Therefore, data describing why screened patients were deemed ineligible are not reported for the trial.

All 250 control arm participants received the referral from the healer for HIV testing, but only 57 presented for HIV testing at a facility. In the 90-day follow up phone call, control arm participants who did not receive an HIV test were asked to describe the major barriers to testing. Participants frequently named more than one barrier to receiving an HIV test through existing resources. The most frequent reason cited for not receiving a test was not having time to go for HIV testing (N=150 of 193 individuals [78%] who did not receive an HIV test within 90 days of study enrollment). The next most common reason for not receiving an HIV test was lack of funds to pay for transportation to the HIV clinic (N=135/193 [70%]). The final reason given for not receiving an HIV test was that the testing facility was perceived as too far away to access (N=8/193 [4%]). In contrast to a prior study of a traditional healer referral program[23], no participant who presented to a clinic was denied testing. No unintended adverse events were noted.

Overall KQ-18 and HIV risk scores were not significantly different between study arms, and in multivariate analysis were not significantly associated with uptake of HIV testing among control arm participants (Table 4). Disaggregated responses from the KQ-18 HIV knowledge and HIV risk questionnaires from participants in the control arm are shown in Tables S3 and S4, respectively. Incorrect responses for two KQ-18 items showed significant association with

reduced rates of HIV testing in univariate analysis. Additionally, three items in the HIV risk questionnaire were significantly associated with reduced rates of HIV testing in univariate analysis.

The intracluster correlation coefficient (ICC) was calculated for each study arm, rather than for the study overall. The ICC for the intervention arm was zero, as all participants received an HIV test, therefore there was no variation among the clusters in this arm. In the control arm, the ICC was calculated at 0.275 (95% CI 0.09 – 0.59). Using the mixed model in the control arm, which adjusts for factors significantly associated with the trial's primary outcome, the adjusted ICC was 0.086 (95% CI 0.015 – 0.363).

Additional Qualitative Results

Traditional healers were described as trustworthy providers and considered more confidential locations to receive HIV testing compared with clinical facilities. Healers in the intervention arm reported that Oraquick HIV tests were easy to deliver and interpret. Traditional healers participating in both study arms were enthusiastic to participate in activities to improve knowledge and uptake of HIV testing among their patients. They described feeling that they could have a positive impact on the health of their patients by supporting uptake of HIV testing, either through referral or delivery of HIV testing.

Qualitative results also help to explain findings that predict low HIV testing uptake among participants in the control arm. Those who reported high-risk behaviors declined to seek HIV testing due to “fear” of testing positive. Lack of funds to pay for transportation to the clinics, which were sometimes described as located very far away, was also described as a barrier to receiving an HIV test. Control arm participants enrolled at bonesetter healers reported that musculoskeletal injuries impeded mobility, which made it difficult to access voluntary HIV testing at clinic-based facilities.

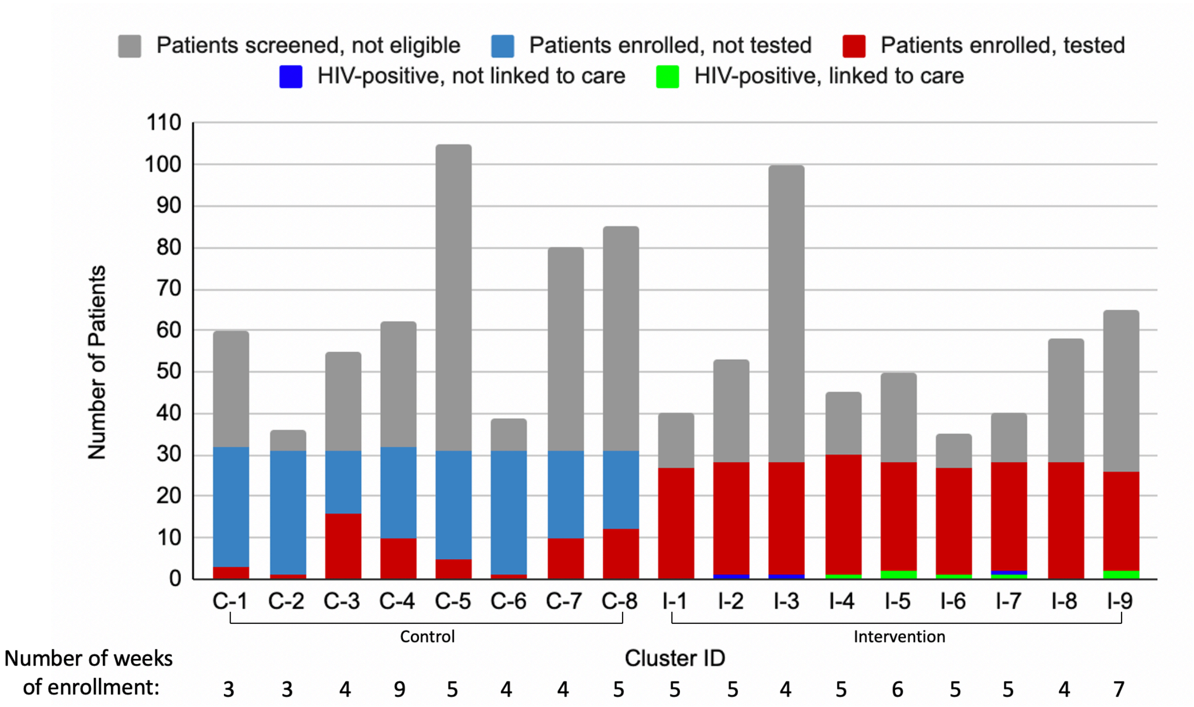

Figure S1. Summary of enrollment and outcomes by study arm and cluster. Numbers of individuals screened (grey), participants enrolled but who did not receive a test (blue), participants enrolled who did receive a test (red). Those testing positive are shown in green (linked to HIV care) and navy (not linked to HIV care). Number of weeks enrolling at each healer site are shown underneath the cluster ID. Cluster ID numbers C-1 through C-8 on the left of the graph reflect control arm sites. Clusters ID numbers I-1 through I-9 on the right of the graph are intervention arm sites.

	Table S1. Study outcomes shown by study arm and by cluster								
**Study Arm**	**Cluster ID**	**Healer type**	**Distance from HIV clinic (in kilometers)**	**Length of enrollment at cluster (in weeks)**	**Patients screened**	**Patients enrolled (% of screened)**	**Patients tested (% of enrolled)**	**Patients HIV-positive (%)**	**HIV-positive linked to care (%)**
Control	1	Herbalist	4.5	3	60	32 (53%)	3 (9%)	0	N/A
	2	Bonesetter	7.5	3	35	31 (88%)	1 (3%)	0	N/A
	3	Herbalist	8	4	55	31 (56%)	16 (52%)	0	N/A
	4	Birth Attendant	5	9	62	32 (52%)	10 (31%)	0	N/A
	5	Spiritualist	5	5	105	31 (30%)	5 (16%)	0	N/A
	6	Bonesetter	8	4	39	31 (79%)	1 (3%)	0	N/A
	7	Spiritualist	5.5	4	80	31 (39%)	10 (32%)	0	N/A
	8	Spiritualist	2.5	5	85	31 (36%)	12 (39%)	0	N/A
	Overall				*486*	*250 (51%)*	*57 (23%)*	*0*	*N/A*
Intervention	1	Spiritualist	7.5	5	40	27 (68%)	27 (100%)	0	N/A
	2	Herbalist	2.5	5	53	28 (53%)	28 (100%)	1 (4%)	0*
	3	Herbalist	3	4	100	28 (28%)	28 (100%)	1 (4%)	0
	4	Herbalist	6.5	5	45	30 (67%)	30 (100%)	1 (3%)	1 (100%)
	5	Birth Attendant	4	6	50	28 (56%)	28 (100%)	2 (7%)	2 (100%)
	6	Bonesetter	4.3	5	35	27 (77%)	27 (100%)	1 (4%)	1 (100%)
	7	Bonesetter	7	5	40	28 (70%)	28 (100%)	2 (7%)	1 (50%)*
	8	Spiritualist	8	4	58	28 (48%)	28 (100%)	0	0
	9	Spiritualist	3.9	7	65	26 (40%)	26 (100%)	2 (8%)	2 (100%)
	Overall				*521*	*250 (48%)*	*250 (100%)*	*10 (4%)*	*7 (70%)*

**Linkage to HIV care could not be established with two newly diagnosed HIV-positive participants who were lost to follow up at 90-days*

Table S2. Characteristics of trial participants newly diagnosed with HIV.					
**Gender**	**Age (years)**	**Marital status**	**# prior HIV tests**	**Months since HIV test**	**Linked to care?**
Female	29	married	0	n/a	LFTU*
Female	28	married	2	21	no
Male	44	married	3	30	yes
Female	34	married	3	21	yes
Male	48	single	2	34	yes
Female	35	married	4	30	yes
Female	38	divorced	8	32	LTFU*
Male	53	married	4	17	yes
Male	38	married	1	57	yes
Female	27	divorced	0	n/a	yes

**"LTFU" denotes participant lost to follow up at 90 days.*

Table S3. Disaggregated results from KQ-18 HIV knowledge questionnaire[7] and univariate analysis for outcome of receiving an HIV test among participants in the control arm (N=250).				
**KQ-18 Item Question**	**Participants responding incorrectly who received HIV testing (N, %)**	**Participants responding correctly who received HIV testing (N, %)**	**Univariate Unadjusted Incidence Rate Ratios, 95% CI**	**P-value**
Coughing and sneezing DO NOT spread HIV	7 (18%)	50 (24%)	0.67 (0.38 – 1.18)	0.17
A person can get HIV by sharing a glass of water with someone who has HIV	15 (35%)	42 (20%)	1.55 (0.99 – 2.44)	0.054
Pulling out the penis before a man climaxes/cums keeps a woman from getting HIV during sex	5 (25%)	52 (23%)	1.00 (0.72 – 1.39)	0.99
A woman can get HIV if she has anal sex with a man	6 (20%)	51 (23%)	0.80 (0.46 – 1.41)	0.45
Showering, or washing one's genitals/ private parts, after sex keeps a person from getting HIV	3 (16%)	54 (23%)	0.69 (0.30 – 1.58)	0.38
All pregnant women infected with HIV will have babies born with AIDS	0 (0%)	57 (25%)	n/a**	0.0095*
People who have been infected with HIV quickly show signs of being infected	10 (21%)	47 (23%)	0.83 (0.56 – 1.25)	0.38
There is a vaccine that can stop adults from getting HIV	19 (22%)	38 (23%)	1.04 (0.68 – 1.59)	0.87
People are likely to get HIV by deep kissing, putting their tongue in their partner's mouth if their partner has HIV	37 (22%)	20 (25%)	0.84 (0.46 – 1.53)	0.57
A woman cannot get HIV if she has sex during her period	3 (27%)	54 (23%)	1.39 (0.49 – 3.92)	0.54
There is a female condom that can help decrease a woman's chance of getting HIV	7 (18%)	50 (24%)	0.76 (0.28 – 2.09)	0.60
A natural skin condom works better against HIV than does a latex condom	56 (24%)	1 (7%)	3.72 (0.65 – 21.4)	0.14
A person will NOT get HIV if she or he is taking antibiotics	4 (13%)	53 (24%)	0.53 (0.31 – 0.91)	0.020*
Having sex with more than one partner can increase a person's chance of being infected with HIV	6 (14%)	51 (25%)	0.58 (0.30 – 1.12)	0.10
Taking a test for HIV one week after having sex will tell a person if she or he has HIV	14 (22%)	43 (23%)	0.86 (0.59 – 1.25)	0.43
A person can get HIV from oral sex	15 (29%)	42 (21%)	1.51 (0.71 – 3.24)	0.29
Using Vaseline or baby oil with condoms lowers the chance of getting HIV	31 (21%)	26 (25%)	0.91 (0.42 – 2.01)	0.82
A person can get HIV by sitting in a hot tub or a swimming pool with a person who has HIV	15 (23%)	42 (23%)	0.92 (0.31 – 2.72)	0.87

**denotes statistical significance at p-value <0.05*
Correctly answering the KQ-18 questionnaire item was used as the reference in the univariate model.
** RR was not estimable due to zero count in one category. Of note, 0/20 who incorrectly answered received HIV testing compared with 57/230 participants who correctly answered received HIV testing. P-value was calculated using Fisher's exact test.

Table S4. Disaggregated results from HIV risk score items and univariate analysis of items related to outcome of receiving an HIV test at existing facilities among participants in the control arm (N=250).				
**Risk Score item**	**Participants responding 'yes' who received HIV testing (N, %)**	**Participants responding 'no' who received HIV testing (N, %)**	**Univariate Unadjusted Incidence Rate Ratios, 95% CI**	**P-value**
Had first sexual intercourse before the age of 13	1 (17%)	56 (23%)	0.80 (0.13 – 5.01)	0.82
Had sexual intercourse with four or more persons in their lifetime	15 (17%)	42 (26%)	0.75 (0.57 – 0.98)	0.038*
Used drugs or alcohol prior to last sexual encounter	0 (0%)	57 (24%)	n/a**	0.044*
Did not use condom during last sexual intercourse	42 (22%)	15 (26%)	0.81 (0.51 – 1.27)	0.36
Had sexual intercourse within the prior 3 months	30 (18%)	27 (33%)	0.55 (0.33 – 0.84)	0.0070*

**denotes statistical significance at p-value <0.05*
Answering "no" to the questionnaire item was used as the reference in the univariate model.
** RR was not estimable due to zero count in one category. Of note, 0/13 of respondents with 'yes' answers to the question received HIV testing compared with 57/237 respondents stating 'no' to the question received HIV testing. P-value was calculated using Fisher's exact test.

References

1. Curran GM, Bauer M, Mittman B, Pyne JM, Stetler C. Effectiveness-implementation hybrid designs: combining elements of clinical effectiveness and implementation research to enhance public health impact. Med Care 2012;50(3):217–26. PMID: 22310560; PMCID: PMC3731143.

2. World Health Organization. WHO Traditional Medicine Strategy 2014 – 2023. Available from http://apps.who.int/medicinedocs/pdf/s2297e/s2297e.pdf. Accessed 27 July 2021.

3. Hooft A, Nabukalu D, Mwanga-Amumpaire J, Gardiner MA, Sundararajan R. Factors Motivating Traditional Healer versus Biomedical Facility Use for Treatment of Pediatric Febrile Illness: Results from a Qualitative Study in Southwestern Uganda. Am J Trop Med Hyg 2020;103(1):501–7. PMID: 32458776; PMCID: PMC735644.

4. Nabukalu D, Ponticiello M, Bennett T, Clark S, King R, Mwanga-Amumpaire J, Sundararajan R. Factors associated with HIV testing among traditional healers and their clients in rural Uganda: Results from a cross-sectional study. Int J STD AIDS. 2021 May 12:9564624211015028. doi: 10.1177/09564624211015028. Epub ahead of print. PMID: 33978547; PMCID: pending.

5. Ugandan Bureau of Statistics. Uganda: Demographic and Health Survey 2016. Available from https://dhsprogram.com/pubs/pdf/FR333/FR333.pdf. Accessed 27 July 2021.

6. Centers for Disease Control and Prevention. Centers for Disease Control and Prevention: HIV testing. Available from https://www.cdc.govhivtestingindex.html. Accessed 27 July 2021.

7. Carey MP, Schroder K. Development and psychometric evaluation of the brief HIV knowledge questionnaire (HIV-KQ-18). AIDS Education and Prevention 14:174–84. PMID: 12000234; PMCID: PMC2423729.

8. Centers for Disease Control and Prevention. Youth Behavior Risk Survey: Data summary and trends report (2007 - 2017). httpswww.cdc.govhealthyyouthdatayrbspdftrendsreport.pdf. Accessed 27 July 2021.

9. Wand H, Reddy T, Naidoo S, et al. A Simple Risk Prediction Algorithm for HIV Transmission: Results from HIV Prevention Trials in KwaZulu Natal, South Africa (2002-2012). AIDS Behav 2018;22(1):325–36. PMID: 28470458.

10. Romo ML, George G, Mantell JE, et al. Depression and sexual risk behavior among long-distance truck drivers at roadside wellness clinics in Kenya. PeerJ 2019;7(6):e7253. PMID: 31355055; PMCID: PMC6642802.

11. Hargreaves JR, Krishnaratne S, Mathema H, et al. Individual and community-level risk factors for HIV stigma in 21 Zambian and South African communities: analysis of data from the HPTN071 (PopART) study. AIDS 2018;32(6):783–93. PMID: 29369164; PMCID: PMC 5854764.

12. Sundararajan R, Langa PV, Morshed T, Manuel S. Traditional Healers as Client

Advocates in the HIV endemic Region of Maputo, Mozambique: Results from a qualitative study. SAHARA-J: Journal of Social Aspects of HIV/AIDS. 2021;18(1):77-85. doi: 10.1080/17290376.2021.1909492. PMID: 33902401; PMCID: PMC8081305.

13. Audet CM, Blevins M, Moon TD, et al. HIV/AIDS-related attitudes and practices among traditional healers in Zambézia Province, Mozambique. J Altern Complement Med 2012;18(12):1133–41. PMID: 23171035; PMCID: PMC3513988.

14. Belete W, Deressa T, Feleke A, et al. Evaluation of diagnostic performance of non-invasive HIV self-testing kit using oral fluid in Addis Ababa, Ethiopia: A facility-based cross-sectional study. PLoS ONE 2019;14(1):e0210866. PMID: 30682062; PMCID: PMC6347142.

15. Zachary D, Mwenge L, Muyoyeta M, et al. Field comparison of OraQuick ADVANCE Rapid HIV-1/2 antibody test and two blood-based rapid HIV antibody tests in Zambia. BMC Infect Dis 2012;12(1):183–7. PMID: 22871032; PMCID: PMC3475053.

16. Kurth AE, Cleland CM, Chhun N, et al. Accuracy and Acceptability of Oral Fluid HIV Self-Testing in a General Adult Population in Kenya. AIDS Behav 2016;20(4):870–9. PMID: 26438487; PMCID: PMC4799243.

17. Patton MQ. Qualitative Evaluation and Research Methods. SAGE Publications, Incorporated; 1990.

18. Hsieh H-F, Shannon SE. Three approaches to qualitative content analysis. Qualitative Health Research. 2005 Nov;15(9):1277–88. PMID: 16204405

19. Weber R. Basic Content Analysis. 2nd ed. Newbury Park: Sage Publications; 1985.

20. Ugandan Ministry of Health. Uganda Population-based HIV Impact Assessment (UPHIA) 2016-2017. Available from http://www.afro.who.intsitesdefaultfiles-UPHIAUgandafactsheet.pdf. Accessed 27 July 2021.

21. Hayes RJ, Bennett S. Simple sample size calculation for cluster-randomized trials. Int J Epidemiol 1999;28(2):319–26. PMID: 10342698.

22. Sharma M, Ying R, Tarr G, Barnabas R. Systematic review and meta-analysis of community and facility-based HIV testing to address linkage to care gaps in sub-Saharan Africa. Nature 2015;528(7580):S77–S85. PMID: 26633769; PMCID: PMC4778960.

23. Audet CM, Salato J, Blevins M, Amsalem D, Vermund SH, Gaspar F. Educational intervention increased referrals to allopathic care by traditional healers in three high HIV-prevalence rural districts in Mozambique. PLoS ONE 2013;8(8):e70326–8. PMID: 23936407; PMCID: PMC3731350.

This supplement contains the following items:

1. Original IRB-approved protocol, dated 1 August 2018
2. Final IRB-approved protocol, dated 4 August 2019
3. Summary of changes

Expanding HIV testing among Ugandan Adults who Utilize Traditional Healers: a cluster randomized controlled trial

Version Date 1 August 2018

WCMC IRB number: 18030119105
MUST Ethical application number: 16/01-17
Clinicaltrials.gov NCT #03718871

Study sponsor: National Institute of Mental Health/US National Institutes of Health (K23MH111409)

**Principal Investigator:**	Radhika Sundararajan, MD PhD<br>Assistant Professor<br>Weill Cornell Department of Emergency Medicine and Center for Global Health<br>525 East 68th Street<br>New York NY 10068
**Co-Investigators:**	Juliet Mwanga-Amumpaire, MD<br>Director, Epicentre Research Centre and<br>Associate Professor of Pediatrics<br>Mbarara University of Science and Technology<br>Mbarara, Uganda
	Daniel Fitzgerald MD<br>Director, Weill Cornell Center for Global Health<br>402 East 67th Street<br>New York NY 10068

Table of Contents

ABBREVIATIONS **3**

1. PROTOCOL SUMMARY **4**

2. BACKGROUND AND RATIONALE **5**

3. STUDY DESIGN **6**

3.1 DESIGN 6
3.2 OVERALL APPROACH 6

4. RECRUITMENT AND ENROLLMENT **6**

4.1 INCLUSION AND EXCLUSION CRITERIA 6
4.2 INFORMED CONSENT 7
4.3 SUBJECT ENROLLMENT 7
4.4 SAMPLE SIZE 7

5. STUDY PROCEDURES **7**

5.1 INTERVENTION ARM 7
5.2 CONTROL ARM 8
5.3 FOLLOW UP 8

6. STATISTICAL CONSIDERATIONS **8**

6.1 OUTCOMES 8
6.2 ANALYSIS PLAN 8
6.3 INTERVENTION EVALUATION 8

7. DATA COLLECTION AND MONITORING **9**

7.1 TRAINING AND CONSIDERATIONS 9
7.2 PRIVACY AND CONFIDENTIALITY 9
7.3 DATA SAFETY AND MONITORING 10
7.4 ADVERSE EVENT REPORTING 10
7.5 QUALITY ASSURANCE 11
7.6 EXPECTED OUTCOMES 11

8. HUMAN SUBJECTS **11**

8.1 PROBLEMS ANTICIPATED 11
8.1.2 Psychological risks *11*
8.1.3 HIV test related discomfort *12*
8.1.4 Possibility of false HIV test results *12*
8.1.5 Loss of confidentiality *12*
8.2 Reasonableness of risks *13*
8.3 Obtaining Informed Consent *13*

9. DISSEMINATION AND PUBLICATION **14**

REFERENCES **15**

ABBREVIATIONS

**AIDS**	Acquired immunodeficiency syndrome
**ART**	Antiretroviral therapy
**DSMB**	Date Safety and Monitoring Board
**HIV**	Human immunodeficiency virus
**IRB**	Institutional Review Board
**ISS**	Immune suppression syndrome
**MUST**	Mbarara University of Science and Technology
**NIH**	National Institutes of Health
**NIMR**	National Institute of Medical Research
**PI**	Principal Investigator
**POC**	Point of care
**PrEP**	Pre-exposure prophylaxis
**TH**	Traditional healer
**WCMC**	Weill Cornell Medical College
**WHO**	World Health Organization

1. PROTOCOL SUMMARY

Study Summary: HIV antiretroviral therapy has the potential to dramatically decrease HIV transmission worldwide[1]; yet, a barrier to ending the AIDS epidemic in low-resource settings is the fact that healthcare is largely provided by traditional or spiritual healers rather than biomedical providers[2-4], and there are no strategies in place to identify HIV-infected patients among Traditional Healer patients and link them to HIV care. In order to reach the UNAIDS 90-90-90 benchmarks HIV services must reach marginalized populations in endemic regions[5], such as in southwestern Uganda. Uganda is one of seven sub-Saharan African countries accounting for 90% of all new HIV infections in this region[6]. HIV prevalence is 7.3%, with ~1.5 million people living with HIV/AIDS and 99,000 new infections in 2014[7]. However, only 50% of sexually active Ugandans have *ever* tested for HIV[8,9]. Like much of sub-Saharan Africa, the majority of Ugandans utilize traditional and faith-based healers[10,11]. Healers are more accessible than biomedical providers in resource-poor settings. Use is driven by preference as well as accessibility: healers are considered uniquely qualified to treat afflictions believed incurable by biomedicine[12]. They are respected community leaders who shape clients' utilization of HIV services[4]. Those who utilize traditional healers may present to biomedical facilities late in their illness, or not at all[13].

Study Aim and Overall Study Design: Develop a *POC HIV testing intervention at TH locations using* a cluster randomized study design. This study will be conducted among TH clients at 10 TH practice locations, to be compared with a control group receiving usual TH care. This pilot will offer HIV testing at TH practice sites to 250 TH clients. Primary outcome for this study will be *rates of HIV testing among TH clients*. Secondary outcomes will include i) *number of new HIV diagnoses* among TH clients, ii) *socio-demographic factors that predict HIV testing*, and iii) *percentage of patients with +HIV POC test who successfully link to HIV care in 3 months*.

This novel approach will have significant public health impact by expanding HIV testing and linkage to care, thereby reducing morbidity, mortality, and HIV transmission in a highly endemic region. This approach could be scaled for delivery in other HIV-endemic, medically pluralistic communities. Pilot results will be integrated with data from ongoing research in southwestern Uganda to propose a large-scale, cluster-randomized trial of this community-based HIV testing program in Eastern Africa.

Study Duration: 12 months (July 2019 – June 2020)

Primary Endpoint: Rate of HIV testing among TH clients at 3 months

Secondary Endpoints: i) number of new HIV diagnoses among TH clients at 3 months post enrollment; ii) percentage of patients with +HIV POC test who successfully link to HIV care at 3 months post enrollment and iii) variables that predict HIV testing.

2. BACKGROUND AND RATIONALE

HIV antiretroviral therapy (ART) has the potential to dramatically decrease HIV transmission worldwide[1,2]. UNAIDS has set the goal that 90% of HIV-infected people should be tested and know their status, so they can access life-saving ART[14,15]. In order to reach the UNAIDS benchmarks, HIV testing must reach marginalized populations in endemic regions[16]. Decentralized[5], community-based[4,5] approaches have been promoted as the key to expanding access to HIV care within low-resource settings. However, successful implementation of community-based services requires an understanding of the social and cultural context that influence community engagement with HIV services. Specifically, many HIV endemic regions are also *medically pluralistic* communities, where multiple explanatory frameworks for health and disease co-exist. In these areas, HIV testing and ART clinical care do not occur in isolation; traditional healers are commonly utilized instead of or concurrently with biomedical services[8,9]. Therefore, the success of decentralized, community-based HIV services must be founded upon a thorough understanding of medical pluralism, and engagement with traditional healers as stakeholders in community health.

Uganda is one of seven sub-Saharan African countries accounting for 90% of all new HIV infections in this region[17]. HIV prevalence is 7.3%, with ~1.5 million people living with HIV/AIDS and 99,000 new infections in 2014[6]. However, only 50% of sexually active Ugandans have *ever* tested for HIV[7]. These data indicate poor progress towards the UNAIDS 90-90-90 targets for ending the HIV epidemic[8,9]. *Our study will characterize factors that shape HIV testing behavior among communities with poor biomedical engagement and pilot an intervention to expand access to HIV testing.* Patients prefer to visit traditional healers to treat "African" diseases, believed incurable by biomedicine[16]. Healers in sub-Saharan Africa serve as herbalists, counselors, social workers, spiritual guides, and legal advisors[12]. Pregnant women may prefer healers as home birth attendants over biomedical facility deliveries[10,12,13,18-21]. Healers are authorities on health concerns, and considered more credible sources of health information than healthcare workers or biomedical clinicians[18,20,21]. As higher rates of HIV testing[3,11,13] and improved engagement with HIV care[22] are correlated with regular contact with a health provider, healers are potential partners for HIV-infected clients throughout the care continuum[23-25]. Our study *will investigate feasibility of involving TH in HIV testing and pilot an intervention to expand HIV testing within communities that use traditional medicine.*

An AIDS-free generation cannot be achieved until HIV care is expanded to communities where the burden of disease continues undetected[26-28]. Healers have access to patients and potential influence to promote HIV testing and engagement with ART services. The proposed research holds promise for dramatically expanding HIV testing and early engagement with HIV services to a large population who otherwise may not access biomedical care.

3. STUDY DESIGN

3.1 Design: Previously conducted qualitative work and observational data will inform implementation of a pilot study, offering HIV testing at traditional healer locations in southwestern Uganda. No compensation will be provided for healers in the intervention or control arms of the intervention. We will follow Ugandan National protocols[29] to administer voluntary HIV counseling and testing at healer practices in Mbarara District. Clustering for this trial will occur at the level of healer practices. For the intervention arm, we will recruit 250 clients from ten healer practice locations. For the control arm, we will observe 250 healer clients at ten healer locations undergoing "enhanced standard of care", where healers will participate in a pre-trial educational session describing community HIV resources available for clients, and how to access them. Enrollment will take place from twenty traditional healer practices, over a period of four months. The follow up period will be three months. Primary outcome for this intervention *will be rates of HIV testing among TH clients* (**Figure**).

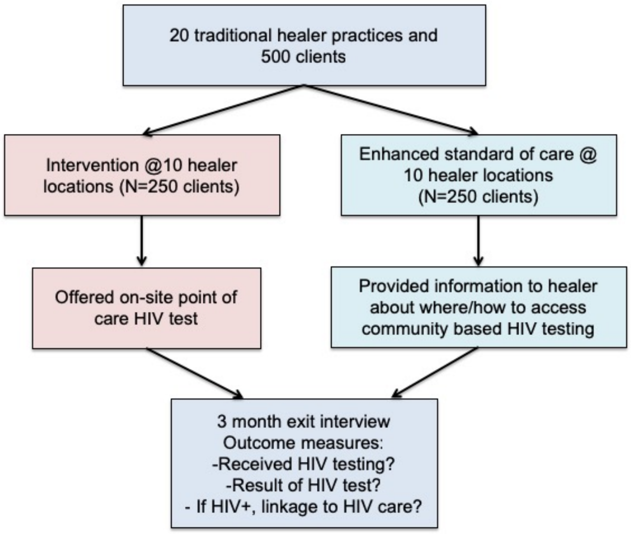

Figure. Approach for HIV testing pilot

3.2 Overall Approach: We will select twenty traditional healer practices to participate in this study from among the ~170 known practices in this District, and group them by specialty (herbalist, birth attendant, spiritualist, bone setter). Within each specialty group, we will randomly select half to participate in the intervention arm (N=10); the remaining half will participate in the control arm (N=10). Healers in both study arms will keep a log of participants indicating whether HIV testing was delivered (intervention) or if the participant was referred to HIV testing (control). Many healers have little formal education and describe themselves as "illiterate"; study logbooks will be created accordingly. Research assistants will create stickers with de-identified study identification numbers for each participant. The healer will place the participant ID sticker in the log book (thereby avoiding the need to be able to write numbers); logs will show pictures for receiving point-of-care HIV testing, or not testing (in the intervention arm), or referral to the District HIV clinic for HIV testing, or not referring (for the control group). Healers will circle the corresponding picture for each participant based on the outcome of the session. Research assistants will be on site, or reachable via mobile phone, to answer any questions the healers have about record keeping or study procedures.

4. RECRUITMENT AND ENROLLMENT

4.1 Inclusion and Exclusion Criteria: Inclusion criteria for healer clients are 1) 18 year of age or older; 2) able to provide informed consent, 3) not known to be HIV infected; 4) willing to be contacted for monthly follow up for 3 months; and 5) willing to complete an exit survey after 3 months. For the intervention (HIV testing) arm, participants must agree to receive their POC HIV test results. Exclusion criteria include being under the age of 18, incapable of giving informed consent, previously being diagnosed with HIV, being unwilling to receive HIV test results, and

unwilling to participate in the testing intervention. Participating healers are eligible to participate if they meet the UNAIDS definition of traditional healer[8], and are willing to participate in study procedures. We will select from among healers with patient volume greater than 7 patients per week in order to ensure that participant recruitment progresses in a timely manner for this pilot study.

4.2 Informed Consent: We will obtain written informed consent from both traditional healers (N=20) and their clients (N=500) participating in this study. Participating clients must consent to providing three telephone contact numbers (self, friend, and family member), home address, and permission for the research team access to medical records from the District HIV clinic. In addition, they must consent to a home visit, if they are not reachable for follow up via phone. Healers must consent to keeping a logbook of which patients received HIV testing (intervention arm) or referred to HIV testing (control arm).

4.3 Subject Enrollment: One research assistant will be assigned to each arm of the study and will base themselves at healer practices to consecutively enroll 25 clients from each healer until we reach as sample size of N=500 (four-month enrollment period). The research assistant will assign a unique study ID number to each participant, collect demographic information, and locator information for the participant and two back up contacts. This information will be used for monthly follow-up assessments, and to deliver the exit interview.

4.4 Sample size: N=250 adults will be enrolled in each arm. Community-based HIV-testing in sub-Saharan Africa has excellent uptake (~97%)[30]; based on these data, we expect 90% uptake of HIV testing in our intervention arm. Assuming HIV prevalence is equivalent to that in our cross-sectional study (9%), we will identify ≥20 newly diagnosed HIV-infected patients among our sample of TH clients ([250 x .90] x 0.09 = 20.25). This aim is exploratory in nature. Sample size will be calculated based on data from an observational census of traditional healers in the study area and will be included in a forthcoming amendment.

5. STUDY PROCEDURES

5.1 Intervention arm: Healers assigned to the intervention arm (N=10) will attend a half-day training session prior to trial initiation, led by District HIV clinic staff. This training session will demonstrate proper use and disposal of the point-of-care HIV testing kit and provide training regarding patient pre- and post-test counseling. Healers will be instructed to discuss the benefits of linking to HIV care, including importance of initiating treatment for HIV infection, reducing possible transmission to others, and health benefits of starting ART early, as part of post-test counseling. In addition, this training session will discuss resources available at the ISS clinic, including mental health counselors, family and partner support groups, counselors trained to discuss serostatus disclosure strategies, and reproductive health counselors. Supplies for point-of-care HIV testing and biohazard-compliant disposal will be provided to healers at the start of the trial. HIV 1/2 antibody point of care test (Oraquick©) will be administered to participants who agree to test. This test is non-invasive, conducted using oral fluids (rather than whole blood), and is FDA-approved for adult use, with results in 20 minutes. Test results are clearly marked with lines, rather than letters or numbers; this test is validated for use by non-medical personnel, with 99.9% specificity and 91.6% sensitivity. Clients with positive tests will be referred to the HIV clinic for Western Blot confirmation, and linkage to care. Following the healer visit, participants will be contacted once per month, for a period of 3 months, to assess for POC test results, and linkage to care for those who test HIV+.

5.2 Control arm: Control arm healers (N=10) will participate in a half-day session prior to trial initiation, led by District HIV clinic staff. This educational session will discuss WHO and Ministry of Health guidelines for HIV testing, who should receive HIV testing and how often, and where to receive it. Participating patients of healers will then receive "enhanced standard of care" as part of the control arm of the trial. Following the visit, study staff will contact the client once per month, for 3 months, to assess for HIV testing since enrollment in the study.

5.3 Follow up: We will contact participants using their mobile phone numbers. If unable to reach after multiple attempts, study staff will call the backup contact to inquire about the participant's (taking care not to mention HIV). If this inquiry is not informative, study staff will visit the participant's home, wearing unmarked clothes and not speaking about HIV with anyone except privately with the participant. In 2014, a Pew Research Center survey indicated that 65% of Ugandans owned a mobile phone (http://www.pewglobal.org/2015/04/15/cell-phones-in-africa-communication-lifeline/); therefore, we anticipate that a majority of participants will have a mobile contact number. If unable to reach the participant at their mobile phone number after multiple attempts, the backup contact will be called. If no mobile number is available for the participant, we will ask for a mobile number for a backup contact to use for communication. Study staff will call the backup contact to attempt to speak with the participant (taking care not to mention HIV). All calls will be made from study cell phones, not linked to any hospital or clinic, such that caller ID or callbacks to the outgoing number will not indicate correlation with HIV research. This process will be followed for participants in both the intervention and control groups. If these inquiries via phone are not informative, a single member of the study staff will visit the participant's home at the address provided, via personal vehicle, wearing unmarked clothes. This staff member will ask to speak with the participant, and will not discuss the study, or the topic of HIV with anyone, except privately with the participant. If home visits are necessary to establish follow-up, we will send a gender-matched staff member (i.e., female staff member for female participant), to minimize suspicion from family or partners who may not be aware of client's participation in the study. This staff member will describe him/herself as a friend from Mbarara, who is inquiring about the whereabouts of the participant.

6. STATISTICAL CONSIDERATIONS

6.1 Outcomes: The primary outcome for this Aim is *rate of HIV testing among TH clients*. Secondary outcomes will include i) *new HIV diagnoses* among TH clients, ii) *patients with +HIV test who successfully link to HIV care in 3 months*[30], and iii) variables that predict HIV testing. The results of this Aim are central to demonstrating feasibility for a subsequent R01 proposal.

6.2 Analysis plan: Descriptive statistics will characterize participants and summarize the data. A multi-level logistic regression model – with individual clients in level 1 nested within healers in level 2 – will be used to calculate the odds ratio for primary outcomes within three months in the intervention compared with the control arms. Adjusted analyses will be conducted as secondary or sensitivity analyses using multivariable, multi-level analyses for both binary and continuous variables. All analyses will be performed in R software.

6.3 Intervention evaluation: 3 months following enrollment, all participants will be contacted for an exit survey to understand experiences of participation in the study, undergoing HIV testing, or deciding not to test. In-depth interviews will be conducted with participating healers at the close of the intervention to obtain feedback on delivering POC testing at their practice locations and assess how the study may have impacted their beliefs and practices.

7. DATA COLLECTION AND MONITORING

7.1 Training and Considerations: All study staff will receive extensive human subjects/research ethics training, including the need for complete confidentiality and to approach questions with sensitivity and in a supportive manner and to provide appropriate referrals when needed. All interviews, surveys and HIV tests will be conducted in private. Study staff will be trained not to press participants to answer questions or engage in intervention activities that seem to be distressing to them, and sessions are terminated if the participant is too distressed, too fatigued, or too frustrated by the effort. Qualitative interviews will be terminated if the participant is too distressed, too fatigued, or too frustrated by the effort. In the event that significant depression symptoms or suicidality are noted, the staff will be instructed to follow procedures in the safety protocol, which include immediately contacting Dr. Mwanga-Amumpaire to arrange referral to appropriate service agencies at Mbarara Regional Referral Hospital or within the community, and reporting incidents or concerns to Dr. Mwanga-Amumpaire or the US-based PI, Dr. Sundararajan.

7.2 Privacy and Confidentiality: We will safeguard against loss of confidentiality in a number of ways. To minimize risk during the consent process, all recruitment and consent for participation will be done in a private tent, with closed panels. Individuals will be recruited separately and undergo informed consent separately (regardless of whether they arrived at the healer location with a friend or family member). This will reduce risk of loss of confidentiality as well as risk of coercion from others. All interviews, surveys, and POC HIV testing will take place in private locations. Pretest and post-test counseling will similarly take place in private tents, therefore minimizing risk of loss of confidentiality during relaying of HIV testing results. During the follow up period, study stuff will not identify the project as one related to HIV; study staff will contact the participant via mobile phone. They will be instructed not to discuss anything related to the study except with the participant. They may visit the participant's home if unable to contact via phone numbers provided. For this, they will wear unmarked clothes and not speak about HIV or the study with anyone, except in private with the participant.

Loss of confidentiality will also be minimized through de-identification of study data. Only the participant's study ID number will appear in the paper or computer-generated files. The key to participants' code number will be encrypted in a computer file, which will be kept in locked offices at MUST. Only Dr. Sundararajan (PI), Dr. Mwanga-Amumpaire, and the research assistants will be able to unencrypt these computer files. In addition, all study staff will be asked to sign a confidentiality pledge. In signing this pledge, the researcher agrees not to divulge the identity of any study participant outside of the study team, nor discuss the particularities of any participant's story with anyone outside the study team.

Study participants may feel reticent to share information about sexual orientation, and this may be a limitation of our study approach. However, the vast majority of HIV transmission in sub-Saharan Africa occurs through heterosexual intercourse[31], and therefore we do not anticipate that unwillingness to disclose homosexual orientation will negatively impact our data collection or results. Participants will be reminded during questionnaire administration that answers regarding sexual orientation will be kept between study participant and research team, and that numerous mechanisms are being undertaken to safeguard their identity, including de-identification of study data.

For potential participants for the testing intervention arm who presents with sexual partners, we will carry out private, individual recruitment and consent, and request if we could include the partner in the HIV testing process. If permission is granted by the participant, then we will 1)

assess if the partner meets criteria for inclusion as a participant; 2) if s/he meets inclusion criteria, the participant will undergo informed consent; and 3) we will conduct the remainder of the study procedures with both members of the couple present, and participating. If the partner does not meet criteria for inclusion, then the partner will be present for his/her partner's study procedures. If permission is refused, we will continue with pre-test counseling, testing, relaying of POC HIV test results, and post-test counseling in private. We will specifically train our research assistants to conduct post-test counseling that discusses issues specific to serodiscordant couples, including reducing risk of HIV transmission, reproductive issues and efficacy of PrEP. In addition, we will encourage the participant to disclose to his/her partner, and refer them to the HIV clinic, which has counselors who specifically assist clients with serostatus disclosure strategies and has resources for serodiscordant couples. By following a strict strategy of individual, private consent, our study procedures seek to reduce coercive influences from partners or family members who may be present with the participant.

MUST has completed numerous research studies on human subjects and maintained full confidentiality of participant records over the preceding decade. MUST has close collaborative relationships with the local HIV clinic, with no-cost resources for testing, treatment and counseling; participants will be referred to these resources as necessary over the course of this research. Due to the nature of this research, we will ensure that study staff undergo training on good clinical practices for human subjects research, with extensive piloting of informed consent and study practices, until staff are comfortable and knowledgeable. Specifically, we will train them to identify situations where coercion may be occurring as to avoid placing undue risk on the potential participant. Refresher courses on relevant topics will be carried out as needed, at least once every 6 months.

7.3 Data Safety and Monitoring: No stopping rules have been defined for this project, given the minimal risks to participants. The research procedures are minimally invasive and present low risks to the study participants. Subjects are volunteers and may drop out of the study at any time, without any recourse. They are informed of such during the consent process.

This study is a pilot intervention, so a DSMB is not required. The PI, Dr. Sundararajan, and local Co-Investigators will review study progress during bi-monthly conference calls. MUST and WCMC IRBs will receive reports annually. Additionally, if necessary, we may utilize the services of the Weill Cornell Medical Center's Data Safety and Monitoring Board (DSMB), which has been designed to ensure the safety and welfare of participants and the validity and confidentiality of data. The board's responsibilities include reviewing protocols, informed consent documents, and plans for data safety and monitoring, evaluating the progress of intervention trials, participant risk versus benefit, periodic assessments of data quality and timeliness, and other factors that can affect the safety of study participants. The board makes recommendations to the PI and NIH (e.g., the observed beneficial or adverse effects of the study, interim analysis of efficacy). The WCMC DSMB meets monthly. The DSMB will receive all adverse event reports and will review stopping rules and interim analyses, where applicable. Data analysis will be conducted every 3 months and discussed with the research team. Annual reports will be submitted to the WCMC IRB, MUST IRB and NIMH.

7.4 Adverse Event Reporting: The Principal Investigator, Dr. Sundararajan, and the Weill Cornell Medical Center IRB will be directly responsible for monitoring the security of the data and safety of participants. Project staff will report emotionally distressed participants to Dr. Sundararajan and Dr. Mwanga-Amumpaire. Dr. Mwanga-Amumpaire will be responsible for evaluating and referring the participant to an appropriate agency at MUST or in the community. Drs. Sundararajan and Mwanga-Amumpaire will be responsible for immediately reporting any

breaches of protocol, breakdowns in the consent process, violations of confidentiality of the data, complaints by participants or any serious problems or adverse events. An incident report will be filed with Dr. Sundararajan. Incident reports, serious problems or adverse events will be reported to the WC IRB by Dr. Sundararajan. The IRB will report to NIH and DHHS if warranted. Requests for the use of these data by persons outside of the project will be decided upon by the PI, Dr. Sundararajan and a committee comprised of coinvestigators. The IRB will be informed and any concerns will be addressed before data are released.

As participation in this study involves minimal commitment, we do not anticipate any negative impact from study closure or from the subject voluntarily leaving the study. Study subjects will not be terminated for any specific reason. All potentially adverse events or serious problems will be reported by Dr. Mwanga-Amumpaire within 48 hours on a standard form and this information will be immediately shared with Dr. Sundararajan for reporting to the Weill Cornell IRB with a copy to the Office of Human Subjects. Study staff will also be debriefed on a weekly basis to address issues that have come up throughout the course of data collection. All project staff will receive verbal and written instructions pertaining to the rules governing the maintenance of confidentiality upon completion of the study or if they leave the study before its completion.

7.5 Quality Assurance: The PIs will oversee data quality control and assurance. They will review all informed consent documents and study eligibility checklist forms. Dr. Sundararajan and Dr. Mwanga-Amumpairewill review data files for internal validity and completeness and send queries to the data management team in Mbarara each week. The team will respond to queries within one week. During the trial, all informed consent and data collection documents will be verified by the study research coordinator.

7.6 Expected Outcomes: The goal of the proposed research is to expand access and engagement with HIV testing in Mbarara by delivering point of care tests at traditional healer locations. This study also has important benefits to healthcare delivery in Uganda as it focuses specifically on early diagnosis of HIV, the leading cause of hospital admissions in this country. The primary and secondary outcomes of the proposed intervention seek to improve health in these regions such that morbidity and mortality are decreased as a result of linkage to outpatient care. The proposed study has the potential to improve rates of HIV testing throughout Uganda, reduce HIV transmission and improve linkage to care. People from other African countries who utilize the services of traditional healers may benefit in the future from the knowledge gained in this study. The results of this study may be expanded to improve access to HIV testing among adults in Uganda and other similar settings. Outcomes will be used to better understand factors influencing engagement with HIV services in endemic areas, where traditional medicine is commonly used. We also hope that the Community Advisory Board formation will facilitate positive, ongoing relationships between healers, biomedical providers, and patients.

8. HUMAN SUBJECTS

8.1 Problems Anticipated:

8.1.2 Psychological risks: For Aim 1, some participants may find it distressing to discuss past HIV testing behaviors, their healthcare seeking trajectory, or experiences with biomedical providers. All study staff will be trained not press participants to answer questions or discuss information that seem to be distressing to them, and to terminate sessions if the participant is overly distressed, fatigued, or frustrated by the effort. In the event that clinically significant depressive symptoms, suicidality, or other psychiatric conditions are noted, the staff will be

instructed to report any incidents or concerns to Dr. Mwanga-Amumpaire, who will assess the participant, offer needed support or arrange for appropriate referral to a counselor or community service provider. In addition, the PI, and all team members have completed Human Subjects Training and Certification Program or equivalent certification at their institution. Dr. Sundararajan has had experience conducting social science research among international, marginalized populations and received previous training in the ethical issues that may arise, and procedures utilized to handle protection against risks. Dr. Mwanga-Amumpaire has years of experience conducting confidential and interviews regarding sensitive topics among socially marginalized and key populations. All study staff will receive extensive human subjects/research ethics training, including the need for complete confidentiality and to approach questions with sensitivity and in a supportive manner and to provide appropriate referrals when needed. Qualitative interviews will be terminated if the participant is too distressed, too fatigued, or too frustrated by the effort. In the event that significant depression symptoms or suicidality are noted, the staff will be instructed to follow procedures in the safety protocol, which include immediately contacting Dr. Mwanga-Amumpaire to arrange referral to appropriate service agencies at MUST or within the community, and reporting incidents or concerns to the US-based PI, Dr. Sundararajan.

It is possible that participants may be identified by others as receiving HIV testing, including healthcare personnel, or sexual partners, or become the subject of HIV-related stigma through participating in this study. In addition, any time a person receives a new diagnosis of being HIV-infected there is a possibility that they will be exposed to stigma from family, partners and other members in their community; external stigma in these cases may involve rejection, estrangement, loss of employment or harassment[32]. Internal stigma may also develop, resulting in shame, depression, and psychological isolation. For women, being HIV-infected may be associated with higher risk of experiencing intimate partner violence. We will work with healers and HIV clinic staff to educate the community to lessen stigma against HIV. Healers will provide post-test counseling for all participants, which will include resources to support their psychosocial health, and provide resources for partners and family members.

8.1.3 HIV test related discomfort: The Oraquick testing process is non-invasive and produces results based on oral fluid samples. We do not anticipate any physical discomfort as a result of the tests administered in the intervention.

8.1.4 Possibility of false HIV test results: Given the high specificity and sensitivity of the rapid tests (99.9% specificity and 91.6% sensitivity), false positives and negatives are unlikely. Participants with positive rapid HIV tests will be referred to the Mbarara Referral Hospital HIV clinic for confirmatory testing, and where they will have CD4 testing and subsequent clinical evaluation. They will be provided specific instructions on how to get there and what to do when they arrive. Healers in the intervention arm will undergo training prior to trial implementation to ensure they are delivering the point of care tests correctly. We note that these point of care tests have been validated for use among laypersons, with extremely high sensitivity/specificity. Research assistants will work with intervention arm healers every few days to ensure testing procedures are followed. Patients with positive HIV testing will be directed to the Mbarara Referral Hospital HIV clinic for confirmatory testing. Patients with negative HIV tests will be counseled to follow up in 3-6 months for re-testing, as is standard protocol according to Ugandan HIV testing guidelines.

8.1.5 Loss of confidentiality: Though this study will include both HIV-infected and non-infected adults, it is possible that participants may be identified by others, including healthcare personnel, as being HIV infected by participating in this study. We will safeguard against this by

conducting interviews, recruitment, and point-of-care testing in private locations. All subject data will be de-identified using a study identification number consisting of a unique code unrelated to any patient identifying information, as per Good Clinical Practice guidelines. Any identifiers linked to the participant will be secured and only available to study staff, to ensure subject confidentiality. Study data will be stored in a locked room at MUST, and on a password protected, encrypted computer. Access to study data will be limited to study staff only. No personally identifiable information will be available during data coding, organization, analysis, or publication. NIMR computers and servers are protected by firewall protection and are only accessible with a password. Study staff will be trained in Good Clinical Practice regarding maintaining confidentiality. We will provide intensive counseling and education to all participants on the importance of confidentiality. In keeping with Ugandan Ministry of Health policy, MUST and Mbarara Regional Referral Hospital does not divulge individual HIV or other results to any third party.

For monthly follow up in Aim 3, study staff will contact the participant via mobile phone. They will be instructed not to discuss anything related to the study except with the participant. They may visit the participant's home if unable to contact via phone numbers provided. For this, they will wear unmarked clothes and not speak about HIV or the study with anyone, except in private with the participant. Participants will be asked to report any breeches in confidentiality, and these will be reported to the IRBs.

8.2 Reasonableness of risks: The research procedures are minimally invasive and present low risks to the study participants. The possible risks include the possibility that participants will become emotionally distressed during the interview process and the potential loss of confidentiality. We have described procedures for dealing with emotional distress or threats to harm self/others, and for minimizing the risk of loss of confidentiality, if it should arise. Given the minimal risks and the safeguards that will be put in place, we believe that the importance of the knowledge to be gained outweighs the potential risks to the subjects involved.

8.3 Obtaining Informed Consent: Written and verbal informed consent will be completed for each participant. During informed consent, the research assistant and potential participant will find a suitable, private location. All potential participants will be told that information gathered in the study is confidential, voluntary, and they are free to take breaks or terminate their participation at any time. A written copy of the informed consent form will be presented in Runyankole or English, depending on the preference of the potential participant. This form will be reviewed orally, and potential participants given opportunity to ask questions. The research assistant will pause after each section and check for understanding by asking the potential participant to describe critical points in his/her own words. If the potential participant agrees to participate, written consent will be documented via signatures from both the research assistant and participant. For those unable to write, they will be allowed to place a thumbprint or "X", with a witness co-signing to ensure proper methods of informed consent have been followed. Each research aim of the study will have its own consent form. Please see those documents attached to this application. No additional consent is necessary. Traditional Healers are considered to have special roles in their communities, as trusted authorities on health, culture and relationships[33] and should be considered key stakeholders for any community-based interventions. As such, their participation and support of this project is highly regarded within specific cultural context. However, this support does not tacitly provide community consent. We will require individual, informed consent for all participants.

The research assistant will review the consent for orally and pause after each section in the consent form to check for understanding by asking the potential participant to describe critical

points in his/her own words. The research staff who will be responsible for obtaining informed consent will assess whether the potential participant has understood the study and consent form by asking key questions (e.g., "How much time will this take you?"; "What are the possible benefits for you?"). Because it is possible that some participants may be cognitively impaired, or they may not initially understand this second consent process, we will test all potential participants for their comprehension of critical in the consent form. Errors will be corrected, and these potential participants will then be asked if they need further clarification. If, after further attempts to clarify any misunderstandings, we determine that they may not fully comprehend the critical aspects of the study, they will not be enrolled.

The vast majority of participants will be non-English speaking, as the primary language in this region is Runyankole. A written copy of the informed consent form will be presented in Runyankole or English, depending on the preference of the potential participant. This form will be reviewed orally, and potential participants given opportunity to ask questions. In addition, we anticipate a significant proportion of potential participants will have low literacy. For those unable to write, they will be allowed to place a thumbprint or "X", with a witness co-signing to ensure proper methods of informed consent have been followed.

Informed consent documents will be retained in locked filing cabinets and storerooms, accessible only to senior investigators or designated study staff. All study logs are identified by study ID numbers, and names are removed. All computerized databases will only contain study ID numbers. The lists linking study numbers to names are kept separately in a password protected computer, accessible only to PI, Co-Investigator, and Project Manager. Clinicians and counselors have received training on the need to maintain full confidentiality of client data.

9. DISSEMINATION AND PUBLICATION

All collaborators will participate in data analysis and research dissemination. Preliminary results will be presented twice per year to the Community Advisory Board, and to the local academic community through conferences at MUST. Abstracts will be submitted to both international and domestic conferences, and we expect that our results will generate two peer reviewed publications ("HIV testing and traditional healing in northeastern Tanzania: results from a qualitative study", and "Delivering HIV testing in eastern Africa at traditional healer practices: results from a pilot intervention"). Data from this pilot will serve as the foundation for an R01 submission to the National Institute of Mental Health, for September 2020 ("A cluster-randomized trial of HIV testing at traditional healer practices in eastern Africa").

REFERENCES

1. Mills EJ, Bakanda C, Birungi J, et al. Life Expectancy of Persons Receiving Combination Antiretrovial Therapy in Low-Income Countries: A Cohort Analysis from Uganda. Annals of Internal Medicine 2011;155:209–16.

2. Bodeker G, Kabatesi D, King R, Homsy J. A regional task force on traditional medicine and AIDS. The Lancet 2000;355(9211):1284.

3. Homsy J, King R, Balaba D, Kabatesi D. Traditional health practitioners are key to scaling up comprehensive care for HIV/AIDS in sub-Saharan Africa. AIDS 2004;18(12):1723–5.

4. King R, Balaba D, Kaboru B, Kabatesi D, Pharris A, Homsy J. The Role of Traditional Healers in Comprehensive HIV/AIDS AIDS AIDS AIDS Prevention and Care in Africa: Untapped Opportunities. In: Marlink R, Tietelman SJ, editors. From The Ground Up. 2009. p. 301–32.

5. Camlin CS, Seeley J, Viljoen L, et al. Strengthening universal HIV “test-and-treat” approaches with social science research. AIDS 2016;30(6):969–70.

6. Joint United Nations Program on HIV/AIDS (UNAIDS). PREVENTION GAP REPORT. 2016.

7. UNAIDS. The HIV and AIDS Uganda Country Progress Report [Internet]. httpwww.aidsuganda.org. 2015 [cited 2015 Sep 21];Available from: http://www.unaids.org/sites/default/files/country/documents/UGA_narrative_report_2015.pdf

8. D CALP, Knowlton AR. Micro-social structural approaches to HIV prevention: a social ecological perspective. AIDS Care 2006;17(sup1):1–82.

9. Ministry of Health II. Uganda Aids Indicator Survey 2011 [Internet]. 2012 [cited 2015 Dec 30];:1–252. Available from: https://www.usaid.gov/sites/default/files/documents/1860/Uganda_AIDS_Indicator_Survey_2011.pdf

10. World Health Organization. WHO Traditional Medicine Strategy 2014 - 2023 [Internet]. httpapps.who.intmedicinedocspdfsese.pdf. 2013 [cited 2015 Dec 30];:1–74. Available from: http://apps.who.int/medicinedocs/pdf/s2297e/s2297e.pdf

11. King R, Homsy J. Involving traditional healers in AIDS education and counselling in sub-Saharan Africa: a review. AIDS 1997;11 Suppl A:S217–25.

12. Green EC. Sexually transmitted disease, ethnomedicine and health policy in Africa. Social Science & Medicine 1992;35(2):121–30.

13. Bodeker G, Carter G, Burford G, Dvorak-Little M. HIV/AIDS: Traditional Systems of Health Care in the Management of a Global Epidemic. The Journal of Alternative and Complementary Medicine 2006;12(6):563–76.

14. Andersen R, Newman JF. Societal and Individual Determinants of Medical Care Utilization in the United States. The Milbank Memorial Fund Quarterly Health and Society 1973;51(1):95.

15. Gelberg L, Andersen RM, Leake BD. The behavioral model for vulnerable populations: Application to medical care use and outcomes for homeless people. Health Services Research 2005;34(6):1273–302.

16. Joint United Nations Program on HIV/AIDS (UNAIDS). 90-90-90: An ambitious treatment target to help end the AIDS epidemic [Internet]. httpwww.unaids.orgsitesdefaultfilesmediaasset--en.pdf. 2014 [cited 2015 Dec 30];:1–40. Available from: http://www.unaids.org/sites/default/files/media_asset/90-90-90_en_0.pdf

17. Ellman T. Demedicalizing AIDS prevention and treatment in Africa. N Engl J Med 2015;372(4):303–5.

18. Ndyomugyenyi R, Neema S, Magnussen P. The use of formal and informal services for antenatal care and malaria treatment in rural Uganda. Health Policy Plan 1998;13(1):94–102.

19. Morris K. Treating HIV in South Africa–a tale of two systems. The Lancet 2001;357(9263):1190.

20. Bulterys M, Fowler MG, Shaffer N, et al. Role of traditional birth attendants in preventing perinatal transmission of HIV. BMJ 2002;324(7331):222–4.

21. Kyomuhendo GB. Low use of rural maternity services in Uganda: impact of women's status, traditional beliefs and limited resources. Reproductive Health Matters 2003;11(21):16–26.

22. Ojikutu B, Nnaji C, Sithole-Berk J, Bogart LM, Gona P. Barriers to HIV Testing in Black Immigrants to the U.S. J Health Care Poor Underserved 2014;25(3):1052–66.

23. Cabral HJ, Tobias C, Rajabiun S, et al. Outreach program contacts: do they increase the likelihood of engagement and retention in HIV primary care for hard-to-reach patients? AIDS Patient Care STDS 2007;21 Suppl 1(s1):S59–67.

24. Tobias C, Cunningham WE, Cunningham CO, Pounds MB. Making the connection: the importance of engagement and retention in HIV medical care. AIDS Patient Care STDS 2007;21 Suppl 1(s1):S3–8.

25. Nyamathi A, Hanson AY, Salem BE, et al. Impact of a rural village women (Asha) intervention on adherence to antiretroviral therapy in southern India. Nurs Res 2012;61(5):353–62.

26. O'Laughlin KN, Wyatt MA, Kaaya S, Bangsberg DR, Ware NC. How treatment partners help: social analysis of an African adherence support intervention. AIDS Behav [Internet] 2012;16(5):1308–15. Available from: http://www.pubmedcentral.nih.gov/articlerender.fcgi?artid=3354325&tool=pmcentrez&rendertype=abstract

27. Audet CM, Hamilton E, Hughart L, Salato J. Engagement of Traditional Healers and Birth Attendants as a Controversial Proposal to Extend the HIV Health Workforce. Curr HIV/AIDS Rep 2015;12(2):238–45.

28. Moshabela M, Zuma T, Orne-Gliemann J, et al. “It is better to die”: experiences of traditional health practitioners within the HIV treatment as prevention trial communities in rural South Africa (ANRS 12249 TasP trial). AIDS Care 2016;28 Suppl 3(sup3):24–32.

29. Abdool Karim SS. Overcoming Impediments to Global Implementation of Early Antiretroviral Therapy. N Engl J Med 2015;373(9):875–6.

30. Audet CM, Blevins M, Moon TD, et al. HIV/AIDS-related attitudes and practices among traditional healers in Zambézia Province, Mozambique. J Altern Complement Med 2012;18(12):1133–41.

31. Ugandan Ministry of Health. Uganda National Policy Guidelines for HIV Counselling and Testing. httpwww.who.inthivpubguidelinesugandaart.pdf. 2007;:1–41.

32. King R. Collaboration with traditional healers in HIV/AIDS prevention and care in sub-Saharan Africa. httpdata.unaids.orgpublicationsirc-pubjc-tradhealen.pdf. 2000;:1–57.

33. Sharma M, Ying R, Tarr G, Barnabas R. Systematic review and meta-analysis of community and facility-based HIV testing to address linkage to care gaps in sub-Saharan Africa. Nature 2015;528(7580):S77–S85.

Summary of Changes to Protocol

August 4, 2019:
As planned in section 4.4 of the protocol, an observational census of traditional healers was conducted in the study area in 2018.

Changes to the inclusion criteria for traditional healers were submitted as follows:
We predetermined an 8-kilometer one-way travel distance to the ISS HIV clinic as the geographic boundary within which to include traditional healers as recruitment locations for this trial. This distance was established as a walking distance to one or more HIV testing resource are centralized. Twenty-five traditional healers were identified within this area. Traditional healers will be considered eligible for participation as cluster sites if they are 1) were aged 18 years or older; 2) have a practice located within eight kilometers of the ISS clinic; 3) were identified in the 2018 population-level census of traditional healers in Mbarara District; and 4) delivered care to at least 7 patients per week. The final criterion excludes healers with average patient volumes in the lowest quartile in order to ensure that participant recruitment progresses in a timely manner for this pilot study.

Changes to the sample size were submitted as follows:
We assumed a baseline testing rate of 39% among our population based on Ministry of Health population-level data[8]. We predicted that the HIV testing rate would increase by 35% to 74% in the intervention arm. Using intracluster correlation coefficient estimate = 0.2, we calculated that we would need to include a minimum of 16 clusters total, with an average of 30 participants recruited per cluster (total sample size of 480 participants), to detect this difference, with 80% power and alpha = 0.05. We plan to recruit 20 healers as clusters in our study to maximize analytical power. Target enrollment number for clients will be 250 per study arm, divided equally among cluster sites in each study arm.

Secondary outcome of “variables that predict HIV testing” was also removed

Expanding HIV testing among Ugandan Adults who Utilize Traditional Healers: a cluster randomized controlled trial

Version Date August 4, 2019

WCMC IRB number: 18030119105
MUST Ethical application number: 16/01-17

Study sponsor: National Institute of Mental Health/US National Institutes of Health (K23MH111409)

Principal Investigator: Radhika Sundararajan, MD PhD
Assistant Professor
Weill Cornell Department of Emergency Medicine and Center for Global Health
525 East 68th Street
New York NY 10068

Co-Investigators: Juliet Mwanga-Amumpaire, MD
Director, Epicentre Research Centre and Associate Professor of Pediatrics
Mbarara University of Science and Technology
Mbarara, Uganda

Daniel Fitzgerald MD
Director, Weill Cornell Center for Global Health
402 East 67th Street
New York NY 10068

Table of Contents

ABBREVIATIONS 3

1. PROTOCOL SUMMARY 4

2. BACKGROUND AND RATIONALE 5

3. STUDY DESIGN 6

3.1 DESIGN 6
3.2 OVERALL APPROACH 6

4. RECRUITMENT AND ENROLLMENT 6

4.1 INCLUSION AND EXCLUSION CRITERIA 6
4.2 INFORMED CONSENT 7
4.3 SUBJECT ENROLLMENT 7
4.4 SAMPLE SIZE 7

5. STUDY PROCEDURES 7

5.1 INTERVENTION ARM 7
5.2 CONTROL ARM 8
5.3 FOLLOW UP 8

6. STATISTICAL CONSIDERATIONS 8

6.1 OUTCOMES 8
6.2 ANALYSIS PLAN 8
6.3 INTERVENTION EVALUATION 9

7. DATA COLLECTION AND MONITORING 9

7.1 TRAINING AND CONSIDERATIONS 9
7.2 PRIVACY AND CONFIDENTIALITY 9
7.3 DATA SAFETY AND MONITORING 10
7.4 ADVERSE EVENT REPORTING 11
7.5 QUALITY ASSURANCE 11
7.6 EXPECTED OUTCOMES 11

8. HUMAN SUBJECTS 12

8.1 PROBLEMS ANTICIPATED 12
8.1.2 Psychological risks *12*
8.1.3 HIV test related discomfort *12*
8.1.4 Possibility of false HIV test results *12*
8.1.5 Loss of confidentiality *13*
8.2 Reasonableness of risks *13*
8.3 Obtaining Informed Consent *13*

9. DISSEMINATION AND PUBLICATION 14

REFERENCES 15

ABBREVIATIONS

**AIDS**	Acquired immunodeficiency syndrome
**ART**	Antiretroviral therapy
**DSMB**	Date Safety and Monitoring Board
**HIV**	Human immunodeficiency virus
**IRB**	Institutional Review Board
**ISS**	Immune suppression syndrome
**MUST**	Mbarara University of Science and Technology
**NIH**	National Institutes of Health
**NIMR**	National Institute of Medical Research
**PI**	Principal Investigator
**POC**	Point of care
**PrEP**	Pre-exposure prophylaxis
**TH**	Traditional healer
**WCMC**	Weill Cornell Medical College
**WHO**	World Health Organization

1. PROTOCOL SUMMARY

Study Summary: HIV antiretroviral therapy has the potential to dramatically decrease HIV transmission worldwide[1]; yet, a barrier to ending the AIDS epidemic in low-resource settings is the fact that healthcare is largely provided by traditional or spiritual healers rather than biomedical providers[2-4], and there are no strategies in place to identify HIV-infected patients among Traditional Healer patients and link them to HIV care. In order to reach the UNAIDS 90-90-90 benchmarks HIV services must reach marginalized populations in endemic regions[5], such as in southwestern Uganda. Uganda is one of seven sub-Saharan African countries accounting for 90% of all new HIV infections in this region[6]. HIV prevalence is 7.3%, with ~1.5 million people living with HIV/AIDS and 99,000 new infections in 2014[7]. However, only 50% of sexually active Ugandans have *ever* tested for HIV[8,9]. Like much of sub-Saharan Africa, the majority of Ugandans utilize traditional and faith-based healers[10,11]. Healers are more accessible than biomedical providers in resource-poor settings. Use is driven by preference as well as accessibility: healers are considered uniquely qualified to treat afflictions believed incurable by biomedicine[12]. They are respected community leaders who shape clients' utilization of HIV services[4]. Those who utilize traditional healers may present to biomedical facilities late in their illness, or not at all[13].

Study Aim and Overall Study Design: Develop a *POC HIV testing intervention at TH locations using* a cluster randomized study design. This study will be conducted among TH clients at 10 TH practice locations, to be compared with a control group receiving usual TH care. This pilot will offer HIV testing at TH practice sites to 250 TH clients. Primary outcome for this study will be *rates of HIV testing among TH clients*. Secondary outcomes will include i) *number of new HIV diagnoses* among TH clients, ii) *socio-demographic factors that predict HIV testing*, and iii) *percentage of patients with +HIV POC test who successfully link to HIV care in 3 months*.

This novel approach will have significant public health impact by expanding HIV testing and linkage to care, thereby reducing morbidity, mortality, and HIV transmission in a highly endemic region. This approach could be scaled for delivery in other HIV-endemic, medically pluralistic communities. Pilot results will be integrated with data from ongoing research in southwestern Uganda to propose a large-scale, cluster-randomized trial of this community-based HIV testing program in Eastern Africa.

Study Duration: 12 months (July 2019 – June 2020)

Primary Endpoint: Rate of HIV testing among TH clients at 3 months

Secondary Endpoints: i) number of new HIV diagnoses among TH clients at 3 months post enrollment; ii) percentage of patients with +HIV POC test who successfully link to HIV care at 3 months post enrollment.

2. BACKGROUND AND RATIONALE

HIV antiretroviral therapy (ART) has the potential to dramatically decrease HIV transmission worldwide[1,2]. UNAIDS has set the goal that 90% of HIV-infected people should be tested and know their status, so they can access life-saving ART[14,15]. In order to reach the UNAIDS benchmarks, HIV testing must reach marginalized populations in endemic regions[16]. Decentralized[5], community-based[4,5] approaches have been promoted as the key to expanding access to HIV care within low-resource settings. However, successful implementation of community-based services requires an understanding of the social and cultural context that influence community engagement with HIV services. Specifically, many HIV endemic regions are also *medically pluralistic* communities, where multiple explanatory frameworks for health and disease co-exist. In these areas, HIV testing and ART clinical care do not occur in isolation; traditional healers are commonly utilized instead of or concurrently with biomedical services[8,9]. Therefore, the success of decentralized, community-based HIV services must be founded upon a thorough understanding of medical pluralism, and engagement with traditional healers as stakeholders in community health.

Uganda is one of seven sub-Saharan African countries accounting for 90% of all new HIV infections in this region[17]. HIV prevalence is 7.3%, with ~1.5 million people living with HIV/AIDS and 99,000 new infections in 2014[6]. However, only 50% of sexually active Ugandans have *ever* tested for HIV[7]. These data indicate poor progress towards the UNAIDS 90-90-90 targets for ending the HIV epidemic[8,9]. *Our study will characterize factors that shape HIV testing behavior among communities with poor biomedical engagement and pilot an intervention to expand access to HIV testing.* Patients prefer to visit traditional healers to treat “African” diseases, believed incurable by biomedicine[16]. Healers in sub-Saharan Africa serve as herbalists, counselors, social workers, spiritual guides, and legal advisors[12]. Pregnant women may prefer healers as home birth attendants over biomedical facility deliveries[10,12,13,18-21]. Healers are authorities on health concerns, and considered more credible sources of health information than healthcare workers or biomedical clinicians[18,20,21]. As higher rates of HIV testing[3,11,13] and improved engagement with HIV care[22] are correlated with regular contact with a health provider, healers are potential partners for HIV-infected clients throughout the care continuum[23-25]. Our study *will investigate feasibility of involving TH in HIV testing and pilot an intervention to expand HIV testing within communities that use traditional medicine.*

An AIDS-free generation cannot be achieved until HIV care is expanded to communities where the burden of disease continues undetected[26-28]. Healers have access to patients and potential influence to promote HIV testing and engagement with ART services. The proposed research holds promise for dramatically expanding HIV testing and early engagement with HIV services to a large population who otherwise may not access biomedical care.

3. STUDY DESIGN

3.1 Design: Previously conducted qualitative work and observational data will inform implementation of a pilot study, offering HIV testing at traditional healer locations in southwestern Uganda. No compensation will be provided for healers in the intervention or control arms of the intervention. We will follow Ugandan National protocols[29] to administer voluntary HIV counseling and testing at healer practices in Mbarara District. Clustering for this trial will occur at the level of healer practices. For the intervention arm, we will recruit 250 clients from ten healer practice locations. For the control arm, we will observe 250 healer clients at ten healer locations undergoing "enhanced standard of care", where healers will participate in a pre-trial educational session describing community HIV resources available for clients, and how to access them. Enrollment will take place from twenty traditional healer practices, over a period of four months. The follow up period will be three months. Primary outcome for this intervention *will be rates of HIV testing among TH clients* (**Figure**).

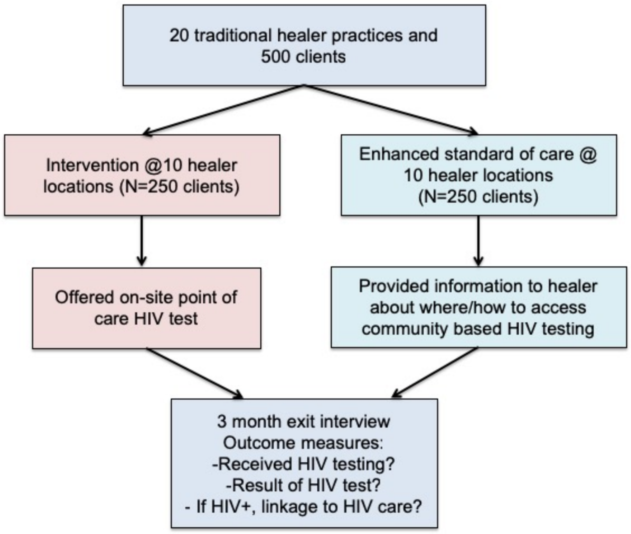

Figure. Approach for HIV testing pilot

3.2 Overall Approach: We will select twenty traditional healer practices to participate in this study from among the ~170 known practices in this District, and group them by specialty (herbalist, birth attendant, spiritualist, bone setter). Within each specialty group, we will randomly select half to participate in the intervention arm (N=10); the remaining half will participate in the control arm (N=10). Healers in both study arms will keep a log of participants indicating whether HIV testing was delivered (intervention) or if the participant was referred to HIV testing (control). Many healers have little formal education and describe themselves as "illiterate"; study logbooks will be created accordingly. Research assistants will create stickers with de-identified study identification numbers for each participant. The healer will place the participant ID sticker in the log book (thereby avoiding the need to be able to write numbers); logs will show pictures for receiving point-of-care HIV testing, or not testing (in the intervention arm), or referral to the District HIV clinic for HIV testing, or not referring (for the control group). Healers will circle the corresponding picture for each participant based on the outcome of the session. Research assistants will be on site, or reachable via mobile phone, to answer any questions the healers have about record keeping or study procedures.

4. RECRUITMENT AND ENROLLMENT

4.1 Inclusion and Exclusion Criteria: Inclusion criteria for healer clients are 1) 18 year of age or older; 2) able to provide informed consent, 3) not known to be HIV infected; 4) willing to be contacted for monthly follow up for 3 months; and 5) willing to complete an exit survey after 3 months. For the intervention (HIV testing) arm, participants must agree to receive their POC HIV test results. Exclusion criteria include being under the age of 18, incapable of giving informed

consent, previously being diagnosed with HIV, being unwilling to receive HIV test results, and unwilling to participate in the testing intervention.

Participating healers are eligible to participate if they meet the UNAIDS definition of traditional healer[8], and are willing to participate in study procedures. We predetermined an 8-kilometer one-way travel distance to the ISS HIV clinic as the geographic boundary within which to include traditional healers as recruitment locations for this trial. This distance was established as a walking distance to one or more HIV testing resource are centralized. Twenty-five traditional healers were identified within this area. Traditional healers will be considered eligible for participation as cluster sites if they are 1) were aged 18 years or older; 2) have a practice located within eight kilometers of the ISS clinic; 3) were identified in the 2018 population-level census of traditional healers in Mbarara District; and 4) delivered care to at least 7 patients per week. The final criterion excludes healers with average patient volumes in the lowest quartile in order to ensure that participant recruitment progresses in a timely manner for this pilot study.

4.2 Informed Consent: We will obtain written informed consent from both traditional healers (N=20) and their clients (N=500) participating in this study. Participating clients must consent to providing three telephone contact numbers (self, friend, and family member), home address, and permission for the research team access to medical records from the District HIV clinic. In addition, they must consent to a home visit, if they are not reachable for follow up via phone. Healers must consent to keeping a logbook of which patients received HIV testing (intervention arm) or referred to HIV testing (control arm).

4.3 Subject Enrollment: One research assistant will be assigned to each arm of the study and will base themselves at healer practices to consecutively enroll clients from each healer until we reach as sample size of N=500 (four-month enrollment period). The research assistant will assign a unique study ID number to each participant, collect demographic information, and locator information for the participant and two back up contacts. This information will be used for monthly follow-up assessments, and to deliver the exit interview.

4.4 Sample size: We assumed a baseline testing rate of 39% among our population based on Ministry of Health population-level data[8]. We predicted that the HIV testing rate would increase by 35% to 74% in the intervention arm. Using intracluster correlation coefficient estimate = 0.2, we calculated that we would need to include a minimum of 16 clusters total, with an average of 30 participants recruited per cluster (total sample size of 480 participants), to detect this difference, with 80% power and alpha = 0.05. We plan to recruit 20 healers as clusters in our study to maximize analytical power. Target enrollment number for clients will be 250 per study arm, divided equally among cluster sites in each study arm.

5. STUDY PROCEDURES

5.1 Intervention arm: Healers assigned to the intervention arm (N=10) will attend a half-day training session prior to trial initiation, led by District HIV clinic staff. This training session will demonstrate proper use and disposal of the point-of-care HIV testing kit and provide training regarding patient pre- and post-test counseling. Healers will be instructed to discuss the benefits of linking to HIV care, including importance of initiating treatment for HIV infection, reducing possible transmission to others, and health benefits of starting ART early, as part of post-test counseling. In addition, this training session will discuss resources available at the ISS clinic, including mental health counselors, family and partner support groups, counselors trained to discuss serostatus disclosure strategies, and reproductive health counselors. Supplies for point-

of-care HIV testing and biohazard-compliant disposal will be provided to healers at the start of the trial. HIV 1/2 antibody point of care test (Oraquick©) will be administered to participants who agree to test. This test is non-invasive, conducted using oral fluids (rather than whole blood), and is FDA-approved for adult use, with results in 20 minutes. Test results are clearly marked with lines, rather than letters or numbers; this test is validated for use by non-medical personnel, with 99.9% specificity and 91.6% sensitivity. Clients with positive tests will be referred to the HIV clinic for Western Blot confirmation, and linkage to care. Following the healer visit, participants will be contacted once per month, for a period of 3 months, to assess for POC test results, and linkage to care for those who test HIV+.

5.2 Control arm: Control arm healers (N=10) will participate in a half-day session prior to trial initiation, led by District HIV clinic staff. This educational session will discuss WHO and Ministry of Health guidelines for HIV testing, who should receive HIV testing and how often, and where to receive it. Participating patients of healers will then receive “enhanced standard of care” as part of the control arm of the trial. Following the visit, study staff will contact the client once per month, for 3 months, to assess for HIV testing since enrollment in the study.

5.3 Follow up: We will contact participants using their mobile phone numbers. If unable to reach after multiple attempts, study staff will call the backup contact to inquire about the participant’s (taking care not to mention HIV). If this inquiry is not informative, study staff will visit the participant’s home, wearing unmarked clothes and not speaking about HIV with anyone except privately with the participant. In 2014, a Pew Research Center survey indicated that 65% of Ugandans owned a mobile phone (http://www.pewglobal.org/2015/04/15/cell-phones-in-africa-communication-lifeline/); therefore, we anticipate that a majority of participants will have a mobile contact number. If unable to reach the participant at their mobile phone number after multiple attempts, the backup contact will be called. If no mobile number is available for the participant, we will ask for a mobile number for a backup contact to use for communication. Study staff will call the backup contact to attempt to speak with the participant (taking care not to mention HIV). All calls will be made from study cell phones, not linked to any hospital or clinic, such that caller ID or callbacks to the outgoing number will not indicate correlation with HIV research. This process will be followed for participants in both the intervention and control groups. If these inquiries via phone are not informative, a single member of the study staff will visit the participant’s home at the address provided, via personal vehicle, wearing unmarked clothes. This staff member will ask to speak with the participant, and will not discuss the study, or the topic of HIV with anyone, except privately with the participant. If home visits are necessary to establish follow-up, we will send a gender-matched staff member (i.e., female staff member for female participant), to minimize suspicion from family or partners who may not be aware of client’s participation in the study. This staff member will describe him/herself as a friend from Mbarara, who is inquiring about the whereabouts of the participant.

6. STATISTICAL CONSIDERATIONS

6.1 Outcomes: The primary outcome for this Aim is *rate of HIV testing among TH clients*. Secondary outcomes will include i) *new HIV diagnoses* among TH clients, and ii) *patients with +HIV test who successfully link to HIV care in 3 months*[30]. The results of this Aim are central to demonstrating feasibility for a subsequent R01 proposal.

6.2 Analysis plan: Descriptive statistics will characterize participants and summarize the data. A multi-level logistic regression model – with individual clients in level 1 nested within healers in level 2 – will be used to calculate the odds ratio for primary outcomes within three months in the

intervention compared with the control arms. Adjusted analyses will be conducted as secondary or sensitivity analyses using multivariable, multi-level analyses for both binary and continuous variables. All analyses will be performed in R software.

6.3 Intervention evaluation: 3 months following enrollment, all participants will be contacted for an exit survey to understand experiences of participation in the study, undergoing HIV testing, or deciding not to test. In-depth interviews will be conducted with participating healers at the close of the intervention to obtain feedback on delivering POC testing at their practice locations and assess how the study may have impacted their beliefs and practices.

7. DATA COLLECTION AND MONITORING

7.1 Training and Considerations: All study staff will receive extensive human subjects/research ethics training, including the need for complete confidentiality and to approach questions with sensitivity and in a supportive manner and to provide appropriate referrals when needed. All interviews, surveys and HIV tests will be conducted in private. Study staff will be trained not to press participants to answer questions or engage in intervention activities that seem to be distressing to them, and sessions are terminated if the participant is too distressed, too fatigued, or too frustrated by the effort. Qualitative interviews will be terminated if the participant is too distressed, too fatigued, or too frustrated by the effort. In the event that significant depression symptoms or suicidality are noted, the staff will be instructed to follow procedures in the safety protocol, which include immediately contacting Dr. Mwanga-Amumpaire to arrange referral to appropriate service agencies at Mbarara Regional Referral Hospital or within the community, and reporting incidents or concerns to Dr. Mwanga-Amumpaire or the US-based PI, Dr. Sundararajan.

7.2 Privacy and Confidentiality: We will safeguard against loss of confidentiality in a number of ways. To minimize risk during the consent process, all recruitment and consent for participation will be done in a private tent, with closed panels. Individuals will be recruited separately and undergo informed consent separately (regardless of whether they arrived at the healer location with a friend or family member). This will reduce risk of loss of confidentiality as well as risk of coercion from others. All interviews, surveys, and POC HIV testing will take place in private locations. Pretest and post-test counseling will similarly take place in private tents, therefore minimizing risk of loss of confidentiality during relaying of HIV testing results. During the follow up period, study stuff will not identify the project as one related to HIV; study staff will contact the participant via mobile phone. They will be instructed not to discuss anything related to the study except with the participant. They may visit the participant’s home if unable to contact via phone numbers provided. For this, they will wear unmarked clothes and not speak about HIV or the study with anyone, except in private with the participant.

Loss of confidentiality will also be minimized through de-identification of study data. Only the participant’s study ID number will appear in the paper or computer-generated files. The key to participants’ code number will be encrypted in a computer file, which will be kept in locked offices at MUST. Only Dr. Sundararajan (PI), Dr. Mwanga-Amumpaire, and the research assistants will be able to unencrypt these computer files. In addition, all study staff will be asked to sign a confidentiality pledge. In signing this pledge, the researcher agrees not to divulge the identity of any study participant outside of the study team, nor discuss the particularities of any participant’s story with anyone outside the study team.

Study participants may feel reticent to share information about sexual orientation, and this may be a limitation of our study approach. However, the vast majority of HIV transmission in sub-Saharan Africa occurs through heterosexual intercourse[31], and therefore we do not anticipate that unwillingness to disclose homosexual orientation will negatively impact our data collection or results. Participants will be reminded during questionnaire administration that answers regarding sexual orientation will be kept between study participant and research team, and that numerous mechanisms are being undertaken to safeguard their identity, including de-identification of study data.

For potential participants for the testing intervention arm who presents with sexual partners, we will carry out private, individual recruitment and consent, and request if we could include the partner in the HIV testing process. If permission is granted by the participant, then we will 1) assess if the partner meets criteria for inclusion as a participant; 2) if s/he meets inclusion criteria, the participant will undergo informed consent; and 3) we will conduct the remainder of the study procedures with both members of the couple present, and participating. If the partner does not meet criteria for inclusion, then the partner will be present for his/her partner's study procedures. If permission is refused, we will continue with pre-test counseling, testing, relaying of POC HIV test results, and post-test counseling in private. We will specifically train our research assistants to conduct post-test counseling that discusses issues specific to serodiscordant couples, including reducing risk of HIV transmission, reproductive issues and efficacy of PrEP. In addition, we will encourage the participant to disclose to his/her partner, and refer them to the HIV clinic, which has counselors who specifically assist clients with serostatus disclosure strategies and has resources for serodiscordant couples. By following a strict strategy of individual, private consent, our study procedures seek to reduce coercive influences from partners or family members who may be present with the participant.

MUST has completed numerous research studies on human subjects and maintained full confidentiality of participant records over the preceding decade. MUST has close collaborative relationships with the local HIV clinic, with no-cost resources for testing, treatment and counseling; participants will be referred to these resources as necessary over the course of this research. Due to the nature of this research, we will ensure that study staff undergo training on good clinical practices for human subjects research, with extensive piloting of informed consent and study practices, until staff are comfortable and knowledgeable. Specifically, we will train them to identify situations where coercion may be occurring as to avoid placing undue risk on the potential participant. Refresher courses on relevant topics will be carried out as needed, at least once every 6 months.

7.3 Data Safety and Monitoring: No stopping rules have been defined for this project, given the minimal risks to participants. The research procedures are minimally invasive and present low risks to the study participants. Subjects are volunteers and may drop out of the study at any time, without any recourse. They are informed of such during the consent process.

This study is a pilot intervention, so a DSMB is not required. The PI, Dr. Sundararajan, and local Co-Investigators will review study progress during bi-monthly conference calls. MUST and WCMC IRBs will receive reports annually. Additionally, if necessary, we may utilize the services of the Weill Cornell Medical Center's Data Safety and Monitoring Board (DSMB), which has been designed to ensure the safety and welfare of participants and the validity and confidentiality of data. The board's responsibilities include reviewing protocols, informed consent documents, and plans for data safety and monitoring, evaluating the progress of intervention trials, participant risk versus benefit, periodic assessments of data quality and timeliness, and other factors that can affect the safety of study participants. The board makes

recommendations to the PI and NIH (e.g., the observed beneficial or adverse effects of the study, interim analysis of efficacy). The WCMC DSMB meets monthly. The DSMB will receive all adverse event reports and will review stopping rules and interim analyses, where applicable. Data analysis will be conducted every 3 months and discussed with the research team. Annual reports will be submitted to the WCMC IRB, MUST IRB and NIMH.

7.4 Adverse Event Reporting: The Principal Investigator, Dr. Sundararajan, and the Weill Cornell Medical Center IRB will be directly responsible for monitoring the security of the data and safety of participants. Project staff will report emotionally distressed participants to Dr. Sundararajan and Dr. Mwanga-Amumpaire. Dr. Mwanga-Amumpaire will be responsible for evaluating and referring the participant to an appropriate agency at MUST or in the community. Drs. Sundararajan and Mwanga-Amumpaire will be responsible for immediately reporting any breaches of protocol, breakdowns in the consent process, violations of confidentiality of the data, complaints by participants or any serious problems or adverse events. An incident report will be filed with Dr. Sundararajan. Incident reports, serious problems or adverse events will be reported to the WC IRB by Dr. Sundararajan. The IRB will report to NIH and DHHS if warranted. Requests for the use of these data by persons outside of the project will be decided upon by the PI, Dr. Sundararajan and a committee comprised of coinvestigators._The IRB will be informed and any concerns will be addressed before data are released.

As participation in this study involves minimal commitment, we do not anticipate any negative impact from study closure or from the subject voluntarily leaving the study. Study subjects will not be terminated for any specific reason. All potentially adverse events or serious problems will be reported by Dr. Mwanga-Amumpaire within 48 hours on a standard form and this information will be immediately shared with Dr. Sundararajan for reporting to the Weill Cornell IRB with a copy to the Office of Human Subjects. Study staff will also be debriefed on a weekly basis to address issues that have come up throughout the course of data collection. All project staff will receive verbal and written instructions pertaining to the rules governing the maintenance of confidentiality upon completion of the study or if they leave the study before its completion.

7.5 Quality Assurance: The PIs will oversee data quality control and assurance. They will review all informed consent documents and study eligibility checklist forms. Dr. Sundararajan and Dr. Mwanga-Amumpairewill review data files for internal validity and completeness and send queries to the data management team in Mbarara each week. The team will respond to queries within one week. During the trial, all informed consent and data collection documents will be verified by the study research coordinator.

7.6 Expected Outcomes: The goal of the proposed research is to expand access and engagement with HIV testing in Mbarara by delivering point of care tests at traditional healer locations. This study also has important benefits to healthcare delivery in Uganda as it focuses specifically on early diagnosis of HIV, the leading cause of hospital admissions in this country. The primary and secondary outcomes of the proposed intervention seek to improve health in these regions such that morbidity and mortality are decreased as a result of linkage to outpatient care. The proposed study has the potential to improve rates of HIV testing throughout Uganda, reduce HIV transmission and improve linkage to care. People from other African countries who utilize the services of traditional healers may benefit in the future from the knowledge gained in this study. The results of this study may be expanded to improve access to HIV testing among adults in Uganda and other similar settings. Outcomes will be used to better understand factors influencing engagement with HIV services in endemic areas, where traditional medicine is commonly used. We also hope that the Community Advisory Board

formation will facilitate positive, ongoing relationships between healers, biomedical providers, and patients.

8. HUMAN SUBJECTS

8.1 Problems Anticipated:

8.1.2 Psychological risks: For Aim 1, some participants may find it distressing to discuss past HIV testing behaviors, their healthcare seeking trajectory, or experiences with biomedical providers. All study staff will be trained not press participants to answer questions or discuss information that seem to be distressing to them, and to terminate sessions if the participant is overly distressed, fatigued, or frustrated by the effort. In the event that clinically significant depressive symptoms, suicidality, or other psychiatric conditions are noted, the staff will be instructed to report any incidents or concerns to Dr. Mwanga-Amumpaire, who will assess the participant, offer needed support or arrange for appropriate referral to a counselor or community service provider. In addition, the PI, and all team members have completed Human Subjects Training and Certification Program or equivalent certification at their institution. Dr. Sundararajan has had experience conducting social science research among international, marginalized populations and received previous training in the ethical issues that may arise, and procedures utilized to handle protection against risks. Dr. Mwanga-Amumpaire has years of experience conducting confidential and interviews regarding sensitive topics among socially marginalized and key populations. All study staff will receive extensive human subjects/research ethics training, including the need for complete confidentiality and to approach questions with sensitivity and in a supportive manner and to provide appropriate referrals when needed. Qualitative interviews will be terminated if the participant is too distressed, too fatigued, or too frustrated by the effort. In the event that significant depression symptoms or suicidality are noted, the staff will be instructed to follow procedures in the safety protocol, which include immediately contacting Dr. Mwanga-Amumpaire to arrange referral to appropriate service agencies at MUST or within the community, and reporting incidents or concerns to the US-based PI, Dr. Sundararajan.

It is possible that participants may be identified by others as receiving HIV testing, including healthcare personnel, or sexual partners, or become the subject of HIV-related stigma through participating in this study. In addition, any time a person receives a new diagnosis of being HIV-infected there is a possibility that they will be exposed to stigma from family, partners and other members in their community; external stigma in these cases may involve rejection, estrangement, loss of employment or harassment[32]. Internal stigma may also develop, resulting in shame, depression, and psychological isolation. For women, being HIV-infected may be associated with higher risk of experiencing intimate partner violence. We will work with healers and HIV clinic staff to educate the community to lessen stigma against HIV. Healers will provide post-test counseling for all participants, which will include resources to support their psychosocial health, and provide resources for partners and family members.

8.1.3 HIV test related discomfort: The Oraquick testing process is non-invasive and produces results based on oral fluid samples. We do not anticipate any physical discomfort as a result of the tests administered as part of the intervention.

8.1.4 Possibility of false HIV test results: Given the high specificity and sensitivity of the rapid tests (99.9% specificity and 91.6% sensitivity), false positives and negatives are unlikely. Participants with positive rapid HIV tests will be referred to the Mbarara Referral Hospital HIV clinic for confirmatory testing, and where they will have CD4 testing and subsequent clinical

evaluation. They will be provided specific instructions on how to get there and what to do when they arrive. Healers in the intervention arm will undergo training prior to trial implementation to ensure they are delivering the point of care tests correctly. We note that these point of care tests have been validated for use among laypersons, with extremely high sensitivity/specificity. Research assistants will work with intervention arm healers every few days to ensure testing procedures are followed. Patients with positive HIV testing will be directed to the Mbarara Referral Hospital HIV clinic for confirmatory testing. Patients with negative HIV tests will be counseled to follow up in 3-6 months for re-testing, as is standard protocol according to Ugandan HIV testing guidelines.

8.1.5 Loss of confidentiality: Though this study will include both HIV-infected and non-infected adults, it is possible that participants may be identified by others, including healthcare personnel, as being HIV infected by participating in this study. We will safeguard against this by conducting interviews, recruitment, and point-of-care testing in private locations. All subject data will be de-identified using a study identification number consisting of a unique code unrelated to any patient identifying information, as per Good Clinical Practice guidelines. Any identifiers linked to the participant will be secured and only available to study staff, to ensure subject confidentiality. Study data will be stored in a locked room at MUST, and on a password protected, encrypted computer. Access to study data will be limited to study staff only. No personally identifiable information will be available during data coding, organization, analysis, or publication. NIMR computers and servers are protected by firewall protection and are only accessible with a password. Study staff will be trained in Good Clinical Practice regarding maintaining confidentiality. We will provide intensive counseling and education to all participants on the importance of confidentiality. In keeping with Ugandan Ministry of Health policy, MUST and Mbarara Regional Referral Hospital does not divulge individual HIV or other results to any third party.

For monthly follow up in Aim 3, study staff will contact the participant via mobile phone. They will be instructed not to discuss anything related to the study except with the participant. They may visit the participant’s home if unable to contact via phone numbers provided. For this, they will wear unmarked clothes and not speak about HIV or the study with anyone, except in private with the participant. Participants will be asked to report any breeches in confidentiality, and these will be reported to the IRBs.

8.2 Reasonableness of risks: The research procedures are minimally invasive and present low risks to the study participants. The possible risks include the possibility that participants will become emotionally distressed during the interview process and the potential loss of confidentiality. We have described procedures for dealing with emotional distress or threats to harm self/others, and for minimizing the risk of loss of confidentiality, if it should arise. Given the minimal risks and the safeguards that will be put in place, we believe that the importance of the knowledge to be gained outweighs the potential risks to the subjects involved.

8.3 Obtaining Informed Consent: Written and verbal informed consent will be completed for each participant. During informed consent, the research assistant and potential participant will find a suitable, private location. All potential participants will be told that information gathered in the study is confidential, voluntary, and they are free to take breaks or terminate their participation at any time. A written copy of the informed consent form will be presented in Runyankole or English, depending on the preference of the potential participant. This form will be reviewed orally, and potential participants given opportunity to ask questions. The research assistant will pause after each section and check for understanding by asking the potential participant to describe critical points in his/her own words. If the potential participant agrees to

participate, written consent will be documented via signatures from both the research assistant and participant. For those unable to write, they will be allowed to place a thumbprint or “X”, with a witness co-signing to ensure proper methods of informed consent have been followed. Each research aim of the study will have its own consent form. Please see those documents attached to this application. No additional consent is necessary. Traditional Healers are considered to have special roles in their communities, as trusted authorities on health, culture and relationships[33] and should be considered key stakeholders for any community-based interventions. As such, their participation and support of this project is highly regarded within specific cultural context. However, this support does not tacitly provide community consent. We will require individual, informed consent for all participants.

The research assistant will review the consent for orally and pause after each section in the consent form to check for understanding by asking the potential participant to describe critical points in his/her own words. The research staff who will be responsible for obtaining informed consent will assess whether the potential participant has understood the study and consent form by asking key questions (e.g., “How much time will this take you?”; “What are the possible benefits for you?”). Because it is possible that some participants may be cognitively impaired, or they may not initially understand this second consent process, we will test all potential participants for their comprehension of critical in the consent form. Errors will be corrected, and these potential participants will then be asked if they need further clarification. If, after further attempts to clarify any misunderstandings, we determine that they may not fully comprehend the critical aspects of the study, they will not be enrolled.

The vast majority of participants will be non-English speaking, as the primary language in this region is Runyankole. A written copy of the informed consent form will be presented in Runyankole or English, depending on the preference of the potential participant. This form will be reviewed orally, and potential participants given opportunity to ask questions. In addition, we anticipate a significant proportion of potential participants will have low literacy. For those unable to write, they will be allowed to place a thumbprint or “X”, with a witness co-signing to ensure proper methods of informed consent have been followed.

Informed consent documents will be retained in locked filing cabinets and storerooms, accessible only to senior investigators or designated study staff. All study logs are identified by study ID numbers, and names are removed. All computerized databases will only contain study ID numbers. The lists linking study numbers to names are kept separately in a password protected computer, accessible only to PI, Co-Investigator, and Project Manager. Clinicians and counselors have received training on the need to maintain full confidentiality of client data.

9. DISSEMINATION AND PUBLICATION

All collaborators will participate in data analysis and research dissemination. Preliminary results will be presented twice per year to the Community Advisory Board, and to the local academic community through conferences at MUST. Abstracts will be submitted to both international and domestic conferences, and we expect that our results will generate two peer reviewed publications (“HIV testing and traditional healing in northeastern Tanzania: results from a qualitative study”, and “Delivering HIV testing in eastern Africa at traditional healer practices: results from a pilot intervention”). Data from this pilot will serve as the foundation for an R01 submission to the National Institute of Mental Health, for September 2020 (“A cluster-randomized trial of HIV testing at traditional healer practices in eastern Africa”).

REFERENCES

1. Mills EJ, Bakanda C, Birungi J, et al. Life Expectancy of Persons Receiving Combination Antiretrovial Therapy in Low-Income Countries: A Cohort Analysis from Uganda. Annals of Internal Medicine 2011;155:209–16.

2. Bodeker G, Kabatesi D, King R, Homsy J. A regional task force on traditional medicine and AIDS. The Lancet 2000;355(9211):1284.

3. Homsy J, King R, Balaba D, Kabatesi D. Traditional health practitioners are key to scaling up comprehensive care for HIV/AIDS in sub-Saharan Africa. AIDS 2004;18(12):1723–5.

4. King R, Balaba D, Kaboru B, Kabatesi D, Pharris A, Homsy J. The Role of Traditional Healers in Comprehensive HIV/AIDS AIDS AIDS AIDS Prevention and Care in Africa: Untapped Opportunities. In: Marlink R, Tietelman SJ, editors. From The Ground Up. 2009. p. 301–32.

5. Camlin CS, Seeley J, Viljoen L, et al. Strengthening universal HIV “test-and-treat” approaches with social science research. AIDS 2016;30(6):969–70.

6. Joint United Nations Program on HIV/AIDS (UNAIDS). PREVENTION GAP REPORT. 2016.

7. UNAIDS. The HIV and AIDS Uganda Country Progress Report [Internet]. httpwww.aidsuganda.org. 2015 [cited 2015 Sep 21];Available from: http://www.unaids.org/sites/default/files/country/documents/UGA_narrative_report_2015.pdf

8. D CALP, Knowlton AR. Micro-social structural approaches to HIV prevention: a social ecological perspective. AIDS Care 2006;17(sup1):1–82.

9. Ministry of Health II. Uganda Aids Indicator Survey 2011 [Internet]. 2012 [cited 2015 Dec 30];:1–252. Available from: https://www.usaid.gov/sites/default/files/documents/1860/Uganda_AIDS_Indicator_Survey_2011.pdf

10. World Health Organization. WHO Traditional Medicine Strategy 2014 - 2023 [Internet]. httpapps.who.intmedicinedocspdfsese.pdf. 2013 [cited 2015 Dec 30];:1–74. Available from: http://apps.who.int/medicinedocs/pdf/s2297e/s2297e.pdf

11. King R, Homsy J. Involving traditional healers in AIDS education and counselling in sub-Saharan Africa: a review. AIDS 1997;11 Suppl A:S217–25.

12. Green EC. Sexually transmitted disease, ethnomedicine and health policy in Africa. Social Science & Medicine 1992;35(2):121–30.

13. Bodeker G, Carter G, Burford G, Dvorak-Little M. HIV/AIDS: Traditional Systems of Health Care in the Management of a Global Epidemic. The Journal of Alternative and Complementary Medicine 2006;12(6):563–76.

14. Andersen R, Newman JF. Societal and Individual Determinants of Medical Care Utilization in the United States. The Milbank Memorial Fund Quarterly Health and Society 1973;51(1):95.

15. Gelberg L, Andersen RM, Leake BD. The behavioral model for vulnerable populations: Application to medical care use and outcomes for homeless people. Health Services Research 2005;34(6):1273–302.

16. Joint United Nations Program on HIV/AIDS (UNAIDS). 90-90-90: An ambitious treatment target to help end the AIDS epidemic [Internet]. httpwww.unaids.orgsitesdefaultfilesmediaasset--en.pdf. 2014 [cited 2015 Dec 30];:1–40. Available from: http://www.unaids.org/sites/default/files/media_asset/90-90-90_en_0.pdf

17. Ellman T. Demedicalizing AIDS prevention and treatment in Africa. N Engl J Med 2015;372(4):303–5.

18. Ndyomugyenyi R, Neema S, Magnussen P. The use of formal and informal services for antenatal care and malaria treatment in rural Uganda. Health Policy Plan 1998;13(1):94–102.

19. Morris K. Treating HIV in South Africa–a tale of two systems. The Lancet 2001;357(9263):1190.

20. Bulterys M, Fowler MG, Shaffer N, et al. Role of traditional birth attendants in preventing perinatal transmission of HIV. BMJ 2002;324(7331):222–4.

21. Kyomuhendo GB. Low use of rural maternity services in Uganda: impact of women's status, traditional beliefs and limited resources. Reproductive Health Matters 2003;11(21):16–26.

22. Ojikutu B, Nnaji C, Sithole-Berk J, Bogart LM, Gona P. Barriers to HIV Testing in Black Immigrants to the U.S. J Health Care Poor Underserved 2014;25(3):1052–66.

23. Cabral HJ, Tobias C, Rajabiun S, et al. Outreach program contacts: do they increase the likelihood of engagement and retention in HIV primary care for hard-to-reach patients? AIDS Patient Care STDS 2007;21 Suppl 1(s1):S59–67.

24. Tobias C, Cunningham WE, Cunningham CO, Pounds MB. Making the connection: the importance of engagement and retention in HIV medical care. AIDS Patient Care STDS 2007;21 Suppl 1(s1):S3–8.

25. Nyamathi A, Hanson AY, Salem BE, et al. Impact of a rural village women (Asha) intervention on adherence to antiretroviral therapy in southern India. Nurs Res 2012;61(5):353–62.

26. O'Laughlin KN, Wyatt MA, Kaaya S, Bangsberg DR, Ware NC. How treatment partners help: social analysis of an African adherence support intervention. AIDS Behav [Internet] 2012;16(5):1308–15. Available from: http://www.pubmedcentral.nih.gov/articlerender.fcgi?artid=3354325&tool=pmcentrez&rendertype=abstract

27. Audet CM, Hamilton E, Hughart L, Salato J. Engagement of Traditional Healers and Birth Attendants as a Controversial Proposal to Extend the HIV Health Workforce. Curr HIV/AIDS Rep 2015;12(2):238–45.

28. Moshabela M, Zuma T, Orne-Gliemann J, et al. “It is better to die”: experiences of traditional health practitioners within the HIV treatment as prevention trial communities in rural South Africa (ANRS 12249 TasP trial). AIDS Care 2016;28 Suppl 3(sup3):24–32.

29. Abdool Karim SS. Overcoming Impediments to Global Implementation of Early Antiretroviral Therapy. N Engl J Med 2015;373(9):875–6.

30. Audet CM, Blevins M, Moon TD, et al. HIV/AIDS-related attitudes and practices among traditional healers in Zambézia Province, Mozambique. J Altern Complement Med 2012;18(12):1133–41.

31. Ugandan Ministry of Health. Uganda National Policy Guidelines for HIV Counselling and Testing. httpwww.who.inthivpubguidelinesugandaart.pdf. 2007;:1–41.

32. King R. Collaboration with traditional healers in HIV/AIDS prevention and care in sub-Saharan Africa. httpdata.unaids.orgpublicationsirc-pubjc-tradhealen.pdf. 2000;:1–57.

33. Sharma M, Ying R, Tarr G, Barnabas R. Systematic review and meta-analysis of community and facility-based HIV testing to address linkage to care gaps in sub-Saharan Africa. Nature 2015;528(7580):S77–S85.
